# Vesicle-enriched secretomes alter bacterial competitive abilities and are drivers of evolution in microbial communities

**DOI:** 10.1093/femsec/fiad141

**Published:** 2023-10-26

**Authors:** Omar M Warsi, Lars Gedda, Katarina Edwards, Dan I Andersson

**Affiliations:** Department of Medical Biochemistry and Microbiology, Uppsala University, Uppsala 75123, Sweden; Department of Chemistry-Ångström, Uppsala University, Uppsala 75237, Sweden; Department of Chemistry-Ångström, Uppsala University, Uppsala 75237, Sweden; Department of Medical Biochemistry and Microbiology, Uppsala University, Uppsala 75123, Sweden

**Keywords:** colicins, experimental evolution, membrane vesicles, microbial community, microbial secretion systems, vesicle-enriched secretomes

## Abstract

Microbial membrane vesicles can carry compounds that inhibit bacterial growth, but how they impact the fitness of the vesicle-producing bacterial species and influence community dynamics remain unexplored questions. To address these questions, we examined the effect of vesicle-enriched secretomes (VESs) in different single-species and multi-species systems. Effects of VESs on single-species growth dynamics were determined for nine bacterial species belonging to four genera (*Escherichia, Salmonella, Pseudomonas* and *Bacillus*) in nutrient-rich and poor growth media. Results showed both species-specific and nutrient-dependent effects of the VESs on bacterial growth. The strongest antagonistic effects were observed for VES isolated from the natural isolates of *E. coli*, while those isolated from *P. aeruginosa* PA14 affected the highest number of species. We further demonstrated that these VESs altered the competitive abilities of the species involved in two-species (*S*. Typhimurium LT2 and *S. arizonae*) and three-species systems (*E. coli, S*. Typhimurium LT2 and *B. subtilis*). Finally, using experimental evolution we showed that different bacterial species could rapidly acquire mutations that abrogated the antagonistic effects of VESs. This study demonstrates how VESs can contribute in shaping microbial communities, both by increasing the competitive ability of a given bacterial species and as a driver of genetic adaptation.

## Teaser

Vesicle-enriched secretomes can play a significant role in shaping microbial communities, both by increasing the competitive ability of a given bacterial species and as a driver of genetic adaptation.

## Introduction

**Figure 1.**
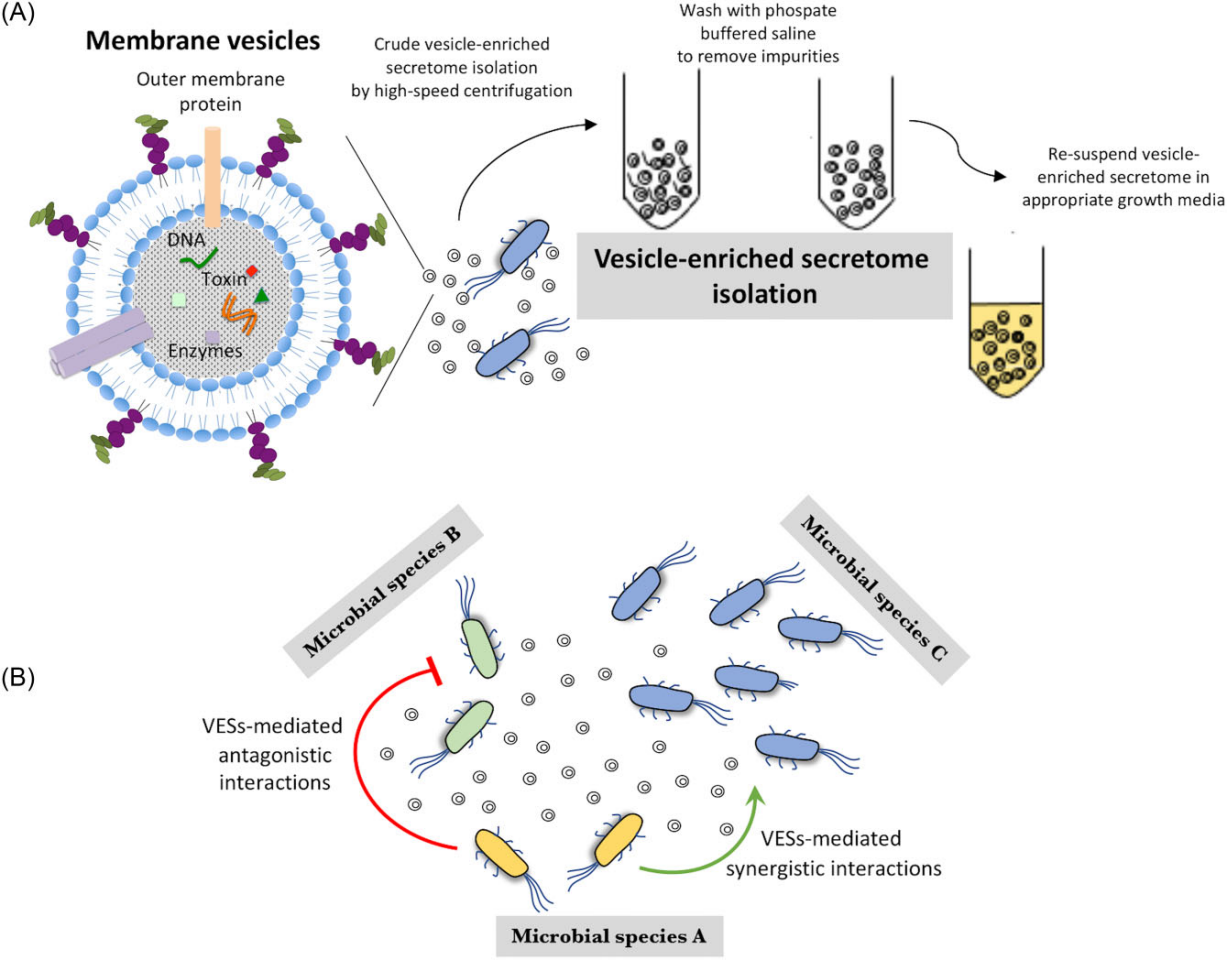
Determining the role of vesicle-enriched secretomes (VESs) on bacterial interactions. (A) Schematic presentation of the preparation of vesicle-enriched secretomes from different bacterial species. The preparations contain membrane vesicles that carry different types of cellular biomolecules. (B) VESs can affect the dynamics of a microbial community by mediating antagonistic and synergistic microbial interactions.

One of the most common ways for bacteria to interact with one another is by secreting different molecules (e.g. nutrients, enzymes, pheromones, or antibacterial agents) in the surrounding environment (Lamont et al. 2002, Nützmann et al. 2011, Traxler et al. 2013). The most well-studied mechanisms employed by bacteria to secrete these molecules in the environment include specific secretion systems (Bleves et al. 2010, Hood et al. 2010, Cianfanelli et al. 2016, Wexler et al. 2016), or in some cases cell lysis (Cavard 2004, Mader et al. 2015). In this study, we determined the role of the secretory system comprised of membrane vesicles in microbial interactions (Fig. 1). Membrane vesicles are spherical bilipid layered structures, 20-250 nm in size, that are released by all bacteria by blebbing out of the membrane and that can secrete different types of molecules (Schwechheimer and Kuehn 2015, Pathirana and Kaparakis-Liaskos 2016, Reidl 2016, Roier et al. 2016, Jan 2017). These vesicles typically carry different types of molecules either within, embedded in the membrane, or bound on the outer surface of the vesicles (Dorward and Garon 1990, Horstman and Kuehn 2000, Roier et al. 2016, Johnston et al. 2023).

Studies that have investigated the association of these vesicles to different bacterial phenotypes have either used purified vesicles (Dorward and Garon 1990, Horstman and Kuehn 2000, Biller et al. 2014, Koeppen et al. 2016) or secretomes that are enriched for vesicles (Kadurugamuwa and Beveridge 1995, 1996, Evans et al. 2012, Koeppen et al. 2016, Zakharzhevskaya et al. 2017, Nice et al. 2018, Johnston et al. 2023) (from now on referred to as vesicle-enriched secretomes or VESs, *see Materials and Methods*), with results from both these approaches offering novel insights about the biological significance of these vesicles. Thus, several studies have shown membrane vesicles and vesicle-enriched secretomes to be important in host-pathogen interactions, and in defense against antibiotics and phages (Kadurugamuwa and Beveridge 1995, Kesty et al. 2004, Kaparakis-Liaskos and Ferrero 2015, Koeppen et al. 2016, Olsen et al. 2016, Zakharzhevskaya et al. 2017, Nice et al. 2018, Reyes-Robles et al. 2018, Balhuizen et al. 2021, Dhital et al. 2022). Although these studies have increased our understanding of the regulation, biogenesis, and medical relevance of the vesicles and vesicle-enriched secretomes, if and how these affect a bacterial's competitive ability and shape microbial communities remain unclear. A few studies investigating the role of vesicles and vesicle-enriched secretomes in microbe-microbe interactions have demonstrated both antagonistic and synergistic effects associated with these vesicles. Important examples of the former are vesicle-enriched secretomes isolated from *Pseudomonas aeruginosa* that were shown to be antagonistic towards both Gram-negative and Gram-positive bacterial species (Kadurugamuwa and Beveridge 1996), and those isolated from the predatory bacterium *Myxococcus xanthus* that carry hydrolytic enzymes with antibacterial activity (Evans et al. 2012). An example of the latter are vesicles isolated from the marine bacterium *Prochlorococcus* that facilitate the growth of other marine bacteria *(Alteromonas and Halomonas)* by acting as a carrier of carbon compounds across the marine microbial community (Biller et al. 2014). Furthermore, membrane vesicles can also induce physiological changes in the surrounding bacteria as was demonstrated in the interactions between *Corynebacterium durum* and *Streptococcus sanguinis*, where the fatty acid containing vesicles from the former induced cell-chain morphology in the latter (Treerat et al. 2020). Vesicles may also aid in extracellular heme cycling as was demonstrated for the Gram-positive bacterium *Dietzia* sp. DQ12-45-1b (Wang et al. 2021). Although these studies demonstrate an association of vesicles to various microbial phenotypes, most of them have only investigated the effects of these vesicles in isolation or in single-species systems.

To examine the relevance of vesicle-enriched secretomes in two- and three-species communities, we investigated how the fitness of a bacterial species changed with different levels of its VESs, and how bacteria adapted and evolved in response to these changes. Such fitness-phenotypes maps have been widely used in evolutionary biology to determine the potential for a trait to evolve under selection and to understand the tempo and mode of adaptive evolution (Dykhuizen et al. 1984, Hartl et al. 1985, Lunzer et al. 2005, Dalziel et al. 2009, Natarajan et al. 2018, Velotta et al. 2018), and are instrumental in highlighting the potential for these vesicles to affect interactions in microbial communities. Our results show that the antagonistic effects of these VESs, depending on bacterial species, could shape the community composition and that bacteria can rapidly evolve to resist these antagonistic effects. These findings suggest that VESs represent an important and unexplored form of selective pressure that can influence microbial communities.

## Materials and methods

### Bacterial strains and culture conditions

Nine different bacterial species were used in this study, including *E. coli* K-12 MG1655, ECOR 5, ECOR 11 and ECOR 36 (the last three are natural isolates of *E. coli* from the ECOR collection), *Salmonella enterica subsp. typhimurium* LT2 (henceforth called *S*. Typhimurium LT2), *Salmonella enterica subsp. arizonae* (henceforth called *S. arizonae*), *Pseudomonas aeruginosa* PA01, *Pseudomonas aeruginosa* PA14 and *Bacillus subtilis*. The nutrient-rich media used in these experiments was Lysogeny broth (LB) and the nutrient-poor media was M9-minimal media containing 0.2% glucose (Leach 1992). All strains were grown at 37°C. For long-term storage of strains, overnight-grown cultures were mixed with DMSO (9 : 1) and were stored at −80°C.

### Isolation of vesicle-enriched secretome (VES)

Vesicle-enriched secretomes were isolated by adapting the methods described previously (McBroom and Kuehn 2007, McMillan and Kuehn 2022). Briefly, each bacterial species was grown in 50 ml of appropriate medium for 16 hrs (bacterial culture densities after 16 hrs of growth is mentioned in Table S1). To obtain a cell-free supernatant 40 ml of the culture was then centrifuged at 4500 r/m for 30 mins. The supernatant was then filtered through a 0.45 µM syringe filter and was then centrifuged again at 40,000 g for 3 hrs. After 3 hrs the supernatant was carefully discarded and the pelleted vesicle-enriched secretomes were washed with phosphate-buffered saline (PBS) once, followed by another round of centrifugation at 40,000 g for 3 hrs. The final pellet was dissolved in 5 ml of the appropriate medium and then filter sterilized again using a 0.45 µM syringe filter. The sterility of the final vesicle preparation was checked by spotting 100 ul on LA plates. The concentrations and size of the vesicles were checked by performing nanoparticle tracking analysis (NTA) (Table S2). NTA was performed on the Nanosight LM10 instrument, which uses a laser beam to illuminate the surface where nanoparticle-sized particles in the suspension can be counted (Malloy and Carr 2006). Each VES was diluted 1 : 100 in phosphate-buffered saline, and the measurement was performed in two 30-seconds frames with the average of these measurements recorded. Two biological replicates were used in each case.

Although different studies have used different speeds of centrifugation to isolate vesicles, we used 40,000 g for 3 hrs, as this has been shown to be sufficient for the isolation of membrane vesicles (McBroom and Kuehn 2007, McMillan and Kuehn 2022). The presence of vesicles in these preparations is confirmed by performing cryo-EM investigation of VESs from ECOR 36 (Fig. S5). Specimen for cryo-EM investigation was prepared at 25°C and high humidity within a custom-built environmental chamber. A small (∼1 µL) drop of the sample was deposited on a carbon-sputtered copper grid (300 mesh, Agar scientific) covered with a perforated polymer film. Excess liquid was thereafter removed by blotting with a filter paper. This leaves a thin film of the solution on the grid. The sample was immediately vitrified in liquid ethane and transferred to the microscope, continuously kept below −160°C, and protected against atmospheric conditions. Analyses were performed with a Zeiss Libra 120 transmission electron microscope (Carl Zeiss AG, Oberkochen, Germany) operating at 80 kV and in zero-loss bright-field mode. Digital images were recorded under low-dose conditions with a BioVision Pro-SM Slow Scan CCD camera (Proscan Elektronische Systeme GmbH, Scheuring, Germany).

The aim of our study was to determine the effects of both the isolated vesicles, as well as of molecules that are enriched with these vesicles, on bacterial interactions. Thus, we only wash the vesicles with PBS to remove any loosely bound components, before re-isolating the washed vesicles by centrifugation at 40,000 g for 3 hrs and do not perform any further purification steps. We refer to these as vesicle-enriched secretomes (VESs). It is possible however that these preparations have other cellular aggregates or cellular components co-isolated as well. On the other hand, the soluble part of the bacterial secretome will not be co-isolated using this protocol.

### Measuring the effect of VESs on growth of different bacterial species

The effect of the vesicle-enriched secretomes on growth of different bacteria was measured by analyzing the growth curves obtained for each bacterial species in the absence and presence of VES. These growth curves were obtained using a BioscreenC analyzer at OD_600_, with measurements taken every 4 min. Overnight grown cultures were diluted 1 : 1000 in an appropriate medium either with or without the VES. 4 biological replicates were used in each case. The exponential growth rate was measured by fitting the OD_600_ values between 0.02 and 0.09 to an exponential growth equation N = N*e^rt^ using the KaleidaGraph software, where r (min^−1^) represents the exponential growth rate. The stationary phase density was calculated using the R-package growthcurver (Sprouffske and Wagner 2016).

### Measuring bacterial fitness in two- and three-species systems

To perform competition experiments in two- and three-species systems, overnight cultures for each strain were mixed in equal ratios (by volume), and the competition was performed in 200 µl of media. In each case, the competition experiments were performed either in the absence or the presence of VESs. The experiment was done over a period of 2 days, with 1 µl of the overnight-grown cultures transferred to 200 µl of the appropriate fresh media (i.e. either with or without VESs) each day. Different concentrations of VESs were added in the competition experiments to determine how these would affect the competitive ability of the less-fit bacteria in the community, where the different concentrations of VESs represent different values of the phenotype of interest. Thus, for the two-species system, 4X- (∼ 8.35E+9 particles/ml) and 8X- (∼1.67E+10 particles/ml) concentrated VESs were used; while for the three-species system, 8X- (∼4.965E+10 particles/ml) concentrated VESs was used. The association between this and the fitness (the competitive ability) of the bacteria would determine the relevance of this phenotype in microbial community interactions. Over the course of the experiment, the frequencies of different strains were measured by tagging different bacteria with genes that encoded fluorescent proteins. In the three species system, *Escherichia coli, Salmonella* Typhimurium LT2 and *Bacillus subtilis* were tagged with yellow, blue and red fluorescent proteins, respectively, and the changes in frequencies of strains over the experiment were measured as changes in the signals of the different fluorescence markers. In the two-species systems, *Salmonella* Typhimurium LT2 was tagged with a yellow fluorescent protein, while *Salmonella arizonae* was untagged, and the change in frequency of strains over the experiment was measured as the changes in the fluorescence to the non-fluorescence signal. The genes encoding the yellow and blue fluorescent proteins were inserted in *E. coli* and *S*. Typhimurium, respectively, using the λ Red system as has been described elsewhere (Elowitz et al. 2002), while the *B. subtilis* strain expressing the red-fluorescent protein was from Ákos T Kovács (Microbiome Ecology at Leiden University, Institute of Biology) (van Gestel et al. 2014). The frequencies of each strain were determined every 24-hrs. This was done by making 100-fold dilutions of cultures from each well in phosphate-buffered saline. 100,000 cells were counted in each case and the fraction of differently labelled cells was determined by flow cytometry (MACSQuant VYB, Miltenyi Biotec). Eight biological replicates were used in each case. The selection coefficient (s) was calculated by plotting natural logs of ratios of population sizes to time, and by calculating the slope of the linearly regressed line such that s = [ln(N_strain1_t_final_/N_strain2_ t_final_)—ln(N_strain1_t_initial_/N_strain2_ t_initial_)]/(t_final-t_initial) (Dykhuizen 1990), where N stands for the population size of the respective strains, while t_initial and t_final are the two time-points where the population sizes were measured.

### Experimental evolution in the presence of VES

To determine the adaptive response of microbial populations towards the antagonistic effects of different VES, we either used *E. coli* K-12 MG1655 or *S*. Typhimurium LT2. The former was serially passaged in the presence of VES isolated from ECOR 11 or ECOR 36, while the latter was serially passaged in the presence of VES isolated from itself. Under each condition, four independent lineages were serially passaged. The evolution experiment was performed in 1 ml culture volume, with 1 µl being transferred every day to the fresh media, and with VESs being added to the media every day. Measurements on growth-rates were done every 30 generations, and the evolved populations were stored at −80°C. The experiments were conducted for one-week, during which time the VES preparations were stored at 4°C.

### Whole-genome sequencing of clones resistant to antagonistic vesicle-enriched secretomes

Clones that had become resistant towards the growth inhibition caused by the VES were whole genome sequenced. DNA was extracted from 1 ml overnight cultures using the MasterPure Complete DNA & RNA Putification Kit (Epicentre) according to the manufacturer's instructions. Illumina's Nextera XT kit was used to make libraries (2×300) that were sequenced on Illumina's Miseq platform. Samples were dual-indexed and pooled together. Average whole genome coverage per sample was ∼30X. Analysis of the fastq files obtained from Miseq sequencing was performed using CLC genomics Workbench version 8 and were mapped to the reference genome of the ancestral *S*. Typhimurium LT2 strain or *E. coli* genome. SNP calling and structural rearrangements were both assessed using this tool.

### Cloning of ColE1 immunity gene

To determine the association between colicins and vesicles, the ColE1 immunity protein was cloned in the IPTG-inducible plasmid pCA24N. Plasmids isolated from ECOR 36 were used as a template to amplify the immunity gene. For the PCR, each primer consisted of a 3′- end corresponding to the annealing site, while 5′-overhangs contained the restriction enzymes sites for *KpnI* and *PstI*. Amplified PCR products were purified using the Qiagen purification kit and then ligated using a T4-ligase. The ligations were then electroporated in *E. coli* K-12 MG1655 and selected on chloramphenicol containing plates. Ten clones were isolated and the presence of the cloned ColE1 gene was confirmed using local Sanger sequencing.

### Statistical analysis

To determine the statistical significance of our data, we either performed an unpaired two-sided Student's t-test or an ANOVA analysis followed by a Tukey's HSD test (post hoc analysis). Thus, the t-test was performed to determine the effect of VESs on the growth of single species of bacteria (Fig. S1), to determine the effect of VESs on three-species competition experiments (Fig. 3), to determine the effect of different nutrient types on VES-mediated microbial interactions (Fig. 6), to determine the association of VESs with different types of colicins (Fig. S3), and to determine the growth differences in mutants resistant to the antagonistic effects of VESs (Figs 4 and 5). Wherever appropriate, Bonferroni's correction was applied to correct for multiple comparisons. The adjusted p-value was obtained by dividing the calculated p-value by the number of comparisons performed.

**Figure 2.**
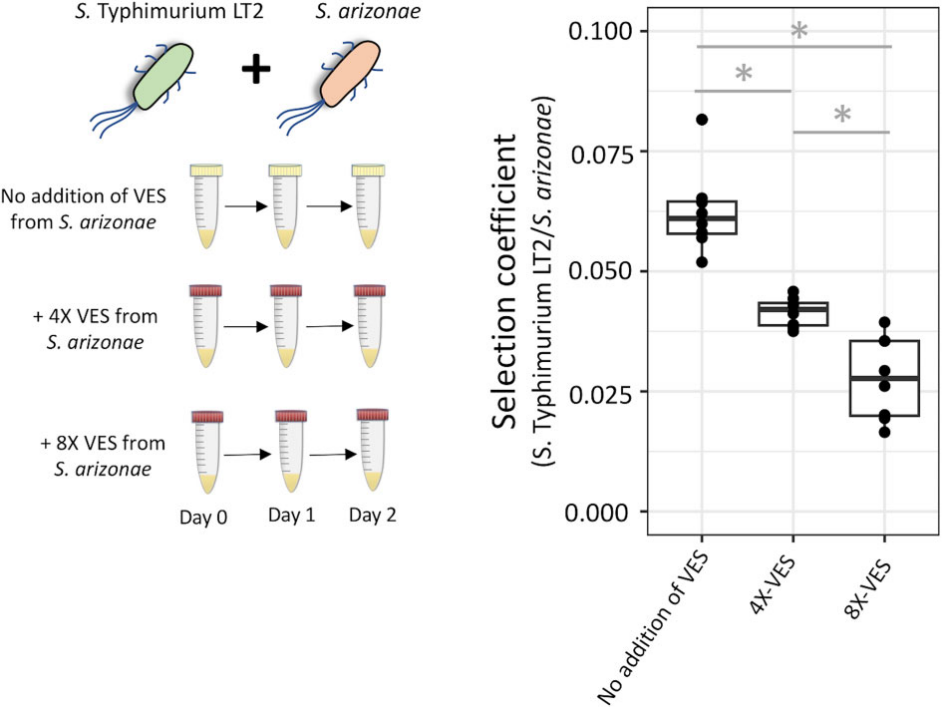
Effect of vesicle-enriched secretome (VES) isolated from *S. arizonae* on competition between *S*. Typhimurium LT2 and *S. arizonae*. Three different concentrations of vesicle-enriched secretomes were used to determine their effect on the competitive ability of *S. arizonae*. The concentration of the 8-fold concentrated VESs used was 1.67E+10 particles/ml. Y-axis shows the selection coefficient measured during competition between *S*. Typhimurium LT2 and S. *arizonae* (*S*. Typhimurium LT2/ *S. arizonae*) in lysogeny broth at 37 °C, while the X-axis shows different concentrations of VES used. Eight replicates were used for each concentration. A one-way ANOVA analysis was performed to determine statistical significance for the effect of VESs on selection coefficients (F=45.88, p= 2.17E-08). This was followed by performing a Tukey's HSD test to determine significant differences between different VES concentrations, where * indicates statistical significance at *P* < 0.01. The median of the data set is represented as a horizontal line.

A one-way ANOVA, followed by a Tukey HSD post hoc analysis, was performed to determine the effect of VESs on two-species competition experiments (the different levels of VESs were considered a factor, Fig. 2) and to determine the role of genetic background on VES-mediated microbial interactions (the different host background were considered a factor, Fig. S4).

All the data and the analysis outputs (including p-values, t-values, F-statistic and adjusted *P*-values) are provided in Tables S3–S6.

## Results

### Effects of VESs on the growth of different species of bacteria

To determine how VESs affect the growth of different species of bacteria in lysogeny broth (LB) growth media, nine different isolates of bacteria (four isolates of *Escherichia coli*, two isolates of *Salmonella enterica*, two isolates of *Pseudomonas aeruginosa*, and one isolate of *Bacillus subtilis*) were studied in the presence and absence of VES isolated from each of the bacteria (Fig. S1). Instead of measuring these effects by normalizing the stationary phase bacterial density (to keep the number of bacterial cells constant across different comparisons) or by normalizing to the number of vesicles produced per bacterial species, these effects were measured by keeping the volume of growth media constant. This was done since both the stationary phase bacterial density and the number of vesicles produced per bacterial species is a function of a given environment, and both might play a role in determining the effect of VESs on bacterial interactions. Thus, we consider keeping the growth environment, volume of growth media, and total growth time constant as the most appropriate setup to determine the relevance of the VES to bacterial interactions.

The concentration of the vesicles used in our assay was measured using the nanoparticle tracking analysis method and ranged from ∼ 10^10–^10^1^ particles/ml (Table S2). In each case, we measured how the VESs affected the exponential growth rate and the stationary phase population density of the bacteria. All the values for these growth parameters are presented relative to values obtained for the growth of the bacteria in the absence of the VESs. To be conservative in our initial screening, we consider only those effects to be biologically relevant that either affected these growth parameters by at least 10%, or those where the relative change in the growth parameters between the presence and absence of VESs was statistically significant at *P* < 0.001 (Two-sided Student's t-test). Our results show that the antagonistic effects of VESs were species-specific (Fig. S1, and Tables S3 and S4). Out of a total of 162 comparisons, the VESs affected the exponential growth rate in 7 cases (5 statistically significant at *P* < 0.001, corrected for multiple testing using Bonferroni's correction, and 2 having an effect of ∼10% or more; the latter includes the effect of VESs of *P. aeruginosa* PA14 on *S. arizonae*, and the effect of VESs of *B. subtilis* on itself; Table S3) and the stationary phase density in 17 cases (12 statistically significant at *P* < 0.001, corrected for multiple testing using Bonferroni's correction, and 5 having an effect of ∼10% or more; the latter include the effect of VESs of *P. aeruginosa* PA14 on *E. coli* K-12 MG1655, the effect of VESs of *B. subtilis* on *S*. Typhimurium LT2, the effect of VESs of *S*. Typhimurium LT2 on *S*. Typhimurium LT2, the effect of VESs of *S. arizonae* on *S*. Typhimurium LT2, and effect of VESs of *P. aeruginosa* PA14 on *S*. Typhimurium LT2. Data is provided in Tables S4). The VES isolated from *P. aeruginosa* PA14 had antagonistic effects on the growth of the highest number of bacterial species tested (seven in total, 3 where exponential growth rate was affected and 4 where stationary phase density was affected, Fig. S1H), while those isolated from natural isolates of *E. coli* i.e. ECOR 11 and ECOR 36 had a strong antagonistic effect on the growth of *E. coli* K-12 MG1655 (Fig. S1C and D). The VESs from *S. arizonae* and *B. subtilis* both displayed antagonistic effects on growth of *S*. Typhimurium LT2 (Fig. S1F and I). Interestingly, *S*. Typhimurium LT2, *E. coli* ECOR 36, and *S. arizonae* all displayed self-inhibition due to their VESs (Fig. S1).

### Fitness-phenotype maps show changes in the competitive ability of bacterial species with changes in levels of VES

We wanted to determine if the VES that displayed antagonistic effects in the single-species experiments would also have similar effects on fitness when the bacterial species were present in multispecies systems. To this end, we generated fitness-phenotype maps for different species of bacteria in two- and three-bacterial species systems, both in the presence and absence of VESs. The two-species system studied was that of *S*. Typhimurium LT2 and *S. arizonae*, and the three-species system was that of *B. subtilis, E. coli* K-12 MG1655 and *S*. Typhimurium LT2. These systems were chosen for two reasons; first, in our single-species experiments (Fig. S1), the VESs from *S. arizonae* were antagonistic towards *S*. Typhimurium LT2, while those isolated from *B. subtilis* were antagonistic towards *S*. Typhimurium LT2. Secondly, in each case VESs were added from the species that was the least fit in two- and three-species competition experiments, i.e. in the two-species system *S. arizonae* was outcompeted by *S*. Typhimurium LT2, while in the three-species system *B. subtilis* was outcompeted by *E. coli* and *S*. Typhimurium LT2.

**Figure 3.**
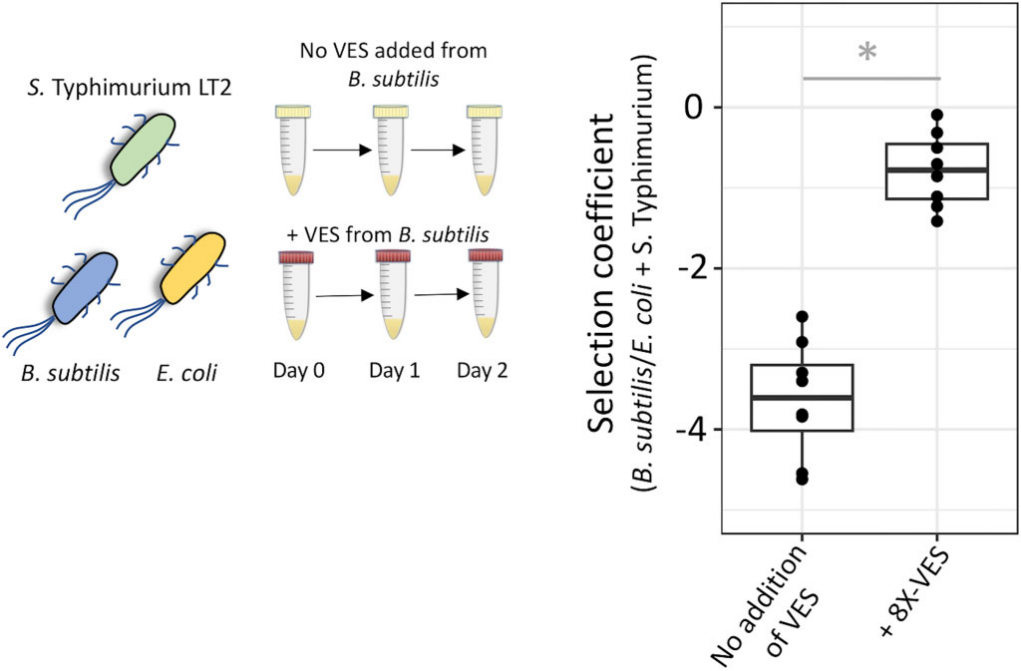
Effect of vesicle-enriched secretome (VES) isolated from *B. subtilis* on competition between *B. subtilis, E. coli* K-12 MG1655 and *S*. Typhimurium LT2. Competition experiments were performed in 3-species systems consisting of *B. subtilis, E. coli* K-12 MG1655 and *S*. Typhimurium LT2 in lysogeny broth at 37°C, in the absence or presence of VES isolated from *B. subtilis*. Y-axis shows the selection coefficient measured during competition between these three strains (*B. subtilis*/*E. coli* + *S*. Typhimurium) while X-axis shows different concentrations of VES used. Eight replicates were used for each concentration. An unpaired two-sided Student's-t test was performed to determine statistical significance and * indicates statistical significance at *P* < 0.001. The median of the data set is represented as a horizontal line. The concentration of the VESs used was 4.965E+10 particles/ml (8-fold concentrated).

In the two-species system, *S*. Typhimurium LT2 outcompeted *S. arizonae* with a selection coefficient of 0.062 ± 0.008 (Fig. 2, Tables S5). Since *S. arizonae* was the less dominant of the two species, we next investigated the outcome of competition between the same two species in the presence of VES isolated from *S. arizonae*. Here, the presence of the VES increased the competitive advantage for *S. arizonae* (Fig. 2). Thus, increasing the concentration of the VES isolated from *S. arizonae* by 4-fold resulted in the selection coefficient reducing from 0.062 ± 0.008 to 0.041 ± 0.003 and by 8-fold resulted in a reduction of the selection coefficient to 0.027 ± 0.008 (statistically significant for one-way ANOVA F=45.88, *P* = 2.17E-08). Statistical significance between the three conditions was further determined by performing a Tukey's HSD (individual *P*-values provided in Table S5). In the three-species bacterial system (*B. subtilis, E. coli* K-12 MG1655 and *S*. Typhimurium LT2), *B. subtilis* was the least dominant bacteria under the conditions tested. Thus, when starting with an initial ratio of 1 : 1 : 1, *B. subtilis* was outcompeted with a selection coefficient of −3.6 ± 0.72 (Fig. 3). However, increasing the concentration of the VES isolated from *B. subtilis* by 8-fold resulted in a drop of the selection coefficient to −0.77 ± 0.46 (t_6.65,14_, *P* = 1.93E-07, Table S5), resulting in its maintenance in the population. These results show that bacterial VESs have the potential to alter the competitive abilities of bacterial species in microbial communities.

### Adaptive responses towards antagonistic effects of VESs

**Figure 4.**
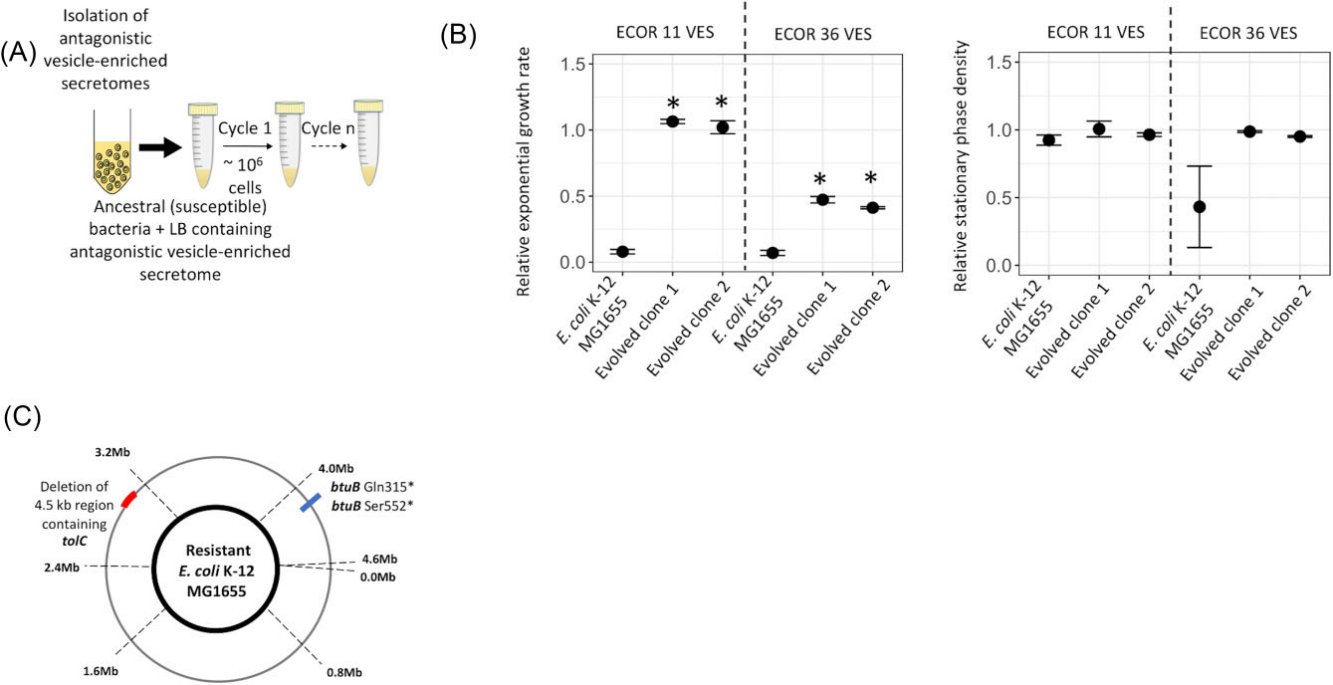
Evolution of *E. coli* K-12 MG1655 populations growing in the presence of antagonistic vesicle-enriched secretomes (VESs). (A) Experimental evolution design for selection of mutants resistant to the antagonistic effects of VESs. The evolution experiment is carried out in lysogeny broth at 37°C, and has VES added to the media at each cycle. (B) Relative exponential growth rate and relative stationary phase density are shown for *E. coli* mutants resistant to antagonistic effects of VES isolated from ECOR 11 and ECOR 36. All growth parameters are shown normalized to values obtained for the susceptible ancestral strain grown in the absence of the VES. Four replicates are used in each case. A two-sided Student's-t test was performed to determine statistical significance and * indicates statistical significance at *P* < 0.001 when comparing the parameters for the ancestral strain to evolved resistant mutants in the presence of vesicle-enriched secretomes. Error bars represent the standard deviation in each case. The concentration of the VESs used in these experiments is 4.16E+10 for VESs isolated from ECOR 11 and 8.375E+10 for VESs isolated from ECOR 36 (Table S2). All experiments were performed in lysogeny broth at 37°C. (C) Mutations were identified in *E. coli* mutants resistant to the antagonistic vesicle-enriched secretomes. Deletions are shown in red, while point mutations are shown in blue.

The single- and multi-species experiments demonstrated that the antagonistic effects of VESs can increase the competitive fitness of vesicle-producing bacterial species. This observation raises the key question of if and how the neighboring bacteria can evolve in response to the antagonistic VESs. To this end, we employed an experimental evolution approach and examined the evolutionary responses to the VESs. We chose the VESs isolated from bacterial species ECOR 11 and ECOR 36 on *E. coli* K-12 MG1655 because they had the largest effect on the growth of other bacterial species (Fig. S1C and D). In each case, the ancestral *E. coli* K-12 MG1655 strain was serially passaged in a growth environment containing the VES from either ECOR 11 or ECOR 36. Evolved clones that were isolated after ∼30 generations of growth displayed resistance toward the antagonistic effects of the VES. The clones that were resistant to the vesicle-enriched secretome isolated from ECOR 11 had a complete recovery of the exponential growth rate (as compared to the ancestral *E. coli* K-12 MG1655 in the presence of the VESs, Fig. 4B and Table S6), while clones resistant to vesicle-enriched secretome isolated from ECOR 36 only had partial recovery of the exponential growth rate (as compared to the ancestral *E. coli* K-12 MG1655 in the presence of the VESs, Fig. 4B and Table S6). The VES isolated from ECOR 11 did not have a strong effect on the stationary phase density of the ancestral *E. coli* K-12 MG1655 (Fig. S1C), and we did not observe any difference between the stationary phase density of the resistant clones as compared to the ancestral strain in the presence of the VES (Fig. 4B and Table S6). On the other hand, VES isolated from ECOR 36 had a strong effect on the stationary phase density of the ancestral strain (relative stationary phase density = 0.43 ± 0.3, Table S6), and the stationary phase density observed for the resistant mutants in the presence of the VES had recovered to values corresponding to that of the ancestral *E. coli* K-12 MG1655 in the absence of the VES (Fig. 4B and Table S6).

To determine the underlying genetic basis for this adaptive response, three resistant clones were whole genome sequenced. Single mutations were observed in two of these clones that in each case resulted in loss of function of the *btuB* gene, while the third clone had a deletion of a ∼4.5 kb region that included the *tolC* gene (Table 1, Fig. 4C). The *btuB* gene encodes a cobalamin outer membrane transporter protein, while *tolC* encodes an outer membrane channel protein that forms a part of the common efflux system of the cell. Of relevance here, both these proteins are also the entry site for colicins that are toxins secreted by different natural isolates of *E. coli*. Both ECOR 11 and ECOR 36 are known colicin producers (Riley and Gordon 1992), carrying the ColE2 and the ColE1 type plasmid, respectively. This suggests that the antagonistic effects of the VES from these natural *E. coli* isolates might be due to the association between the vesicles and colicins. To test this hypothesis, the *colE1* immunity gene that encodes the immunity protein against the colicin ColE1 was cloned on an IPTG-inducible plasmid and the plasmid was then transformed into the susceptible ancestral *E. coli* K-12 MG1655. When the ancestral strain carried the immunity protein it was more resistant to the antagonistic effects of the VES isolated from ECOR 36, as compared to when it carried an empty vector (Fig. S2), confirming that the antagonistic effects of the VESs from the natural isolates of *E. coli* were due to the associations between colicins and the vesicles.

**Table 1.** Mutations identified in *E. coli* K-12 MG1655 mutants resistant to antagonistic effects of membrane vesicle-enriched secretomes isolated from ECOR 11 and ECOR 36.

Strain	Resistant to vesicle-enriched secretome isolated from	Mutation (amino acid change)
DA66942	ECOR 11	*btuB* (Gln315*)
DA66943	ECOR 11	*btuB* (Ser552*)
DA66944	ECOR 36	Deletion of 4.5 kb region including the *tolC* gene

* Indicates a stop codon.

### Different types of colicins are enriched with membrane vesicles in a host-specific manner

To determine whether or not membrane vesicles are enriched with different types of colicins, we isolated the VESs from other colicin-producing *E. coli* strains in the ECOR strain collection. These included ECOR 12 (ColE1), ECOR 14 (Col1a), ECOR 25 (ColB), ECOR 38 (Col1a), ECOR 41 (ColK and/or N), ECOR 42 (ColB), ECOR 48 (Col1b), ECOR 60 (ColE2) and ECOR 62 (ColB). Vesicle-enriched secretomes isolated from all the colicin producers affected growth of *E. coli* K-12 MG1655 (Fig. S3), although to different levels. The strongest effect was observed for the VES isolated from ECOR 41 carrying the colicins ColK and/or N and from ECOR 60 carrying colicins ColE2 (no growth observed in either case), while those isolated from ECOR 25 (ColB) and ECOR 38 (Col1a) had the least effect (relative exponential growth rate of 0.93 ± 0.02 and 0.92 ± 0.04, respectively, and relative stationary phase density of 0.99 ± 0.008 and 0.99 ± 0.005, respectively). Interestingly, even when the ECOR isolates were secreting the same type of colicin, the antagonistic effect of their VES varied. Thus, the VESs of ECOR 36 and ECOR 12 (both expressing the Col E1) and of ECOR 25, ECOR 42, and ECOR 62 (all expressing the ColB) displayed different antagonistic effects (Fig. S4). Among colicin B producing strains, ECOR 42 had the strongest antagonistic effect. The effect of VESs isolated from these strains on relative stationary phase density was statistically different from one another, while only ECOR 42 had a statistically significant effect on the relative exponential growth rate of *E. coli* K-12 MG1655 (Fig. S4; F-values, *P*-values for one-way ANOVA and adjusted p-values for Tukey's HSD given in Tables S3 and S4). Among colicin E1 producing strains, VESs isolated from ECOR 36 had a much stronger and statistically different effect as compared to ECOR 12 (Fig. S4; F-values, *P*-values for one-way ANOVA and adjusted *P*-values for Tukey's HSD given in Tables S3 and S4). These results suggest that the antagonistic effects of the VESs, besides being dependent on the causal protein itself, also depend on the genotype of the bacteria.

### Evolution in response towards self-inhibition from vesicle-enriched secretomes

**Figure 5.**
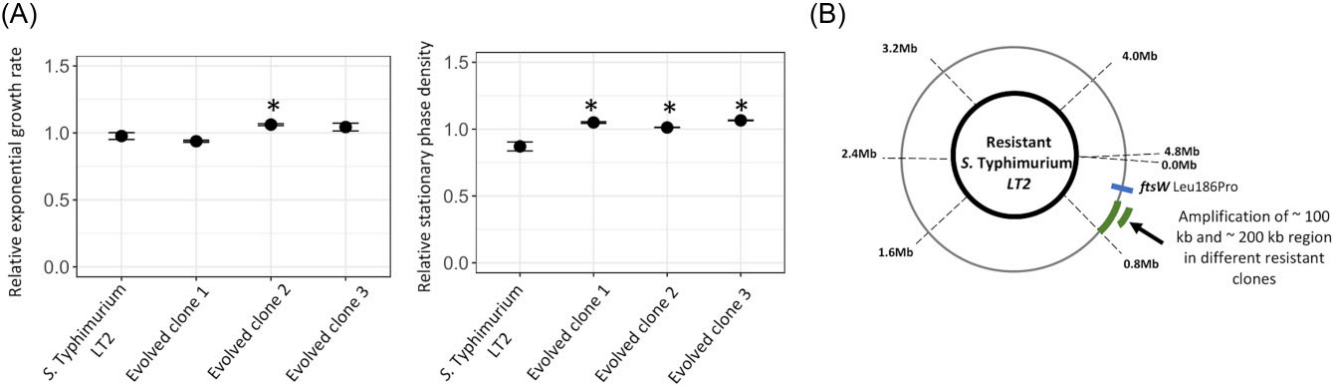
Evolution of *S*. Typhimurium LT2 towards self-inhibition due to antagonistic vesicle-enriched secretomes (VESs). (A) Relative exponential growth rate and relative stationary phase density are shown for *S*. Typhimurium LT2 mutants resistant to self-inhibition by antagonistic VES isolated from *S*. Typhimurium LT2. All growth parameters are shown normalized to values obtained for the susceptible ancestral strain grown in the absence of the VES. For the measurement of relative exponential growth rates and relative stationary phase density, two replicates are used. A two-sided Student's-t test was performed to determine statistical significance and * indicates statistical significance at *P* < 0.001 when comparing the parameters for the ancestral strain to evolved resistant mutants in the presence of vesicle-enriched secretomes. Error bars represent the standard deviation in each case. The concentration of the VESs used is 5.885E+10 particles/ml (Table S2). All experiments were performed in lysogeny broth at 37°C. (B) Mutations identified in *S*. Typhimurium LT2 mutants resistant to the antagonistic vesicle-enriched secretomes. Amplifications are shown in green, and point mutations are shown in blue.

In the initial analysis of the effect of VESs on the growth of single species of bacteria, several cases of self-inhibition were observed. To determine the underlying mechanism for self-inhibition, we investigated the evolutionary responses of *S*. Typhimurium LT2 when it was exposed to its own VES. Evolved clones isolated from populations passaged in the presence of the VESs for ∼70 generations of growth demonstrated resistance towards the self-inhibition (Fig. 5A). All three mutants that were resistant to self-inhibition by the vesicle-enriched secretome had a higher relative stationary phase density (statistically different at *P* < 0.001 for a two-sided Student's t-test; Table S6), while only one of the clones had a higher relative exponential growth rate (statistically different at *P* < 0.001 for a two-sided Student's t-test; Table S6).

To determine the mechanistic basis for this observed reduced susceptibility, we whole genome sequenced three resistant clones (Table 2). In two of the resistant clones, large-scale genome amplifications spanning a 100-kb region in one resistant clone, and a 200-kb region in the other resistant clone were observed. Although these amplifications were observed in overlapping regions of the genome (Fig. 5B, Table 2), given the large number of genes involved within these amplifications the genetic basis of resistance is still unclear in these clones. In the third resistant clone, we observed a non-synonymous mutation in the gene *ftsW* (Leu186Pro). Since this was the only mutation found in this clone, it directly links the protein FtsW in conferring resistance to self-inhibition caused by the VES. The *ftsW* gene codes for an essential cell-division protein that functions as a peptidoglycan glycosyltransferase. This suggests that the self-inhibitory effect of the VESs observed in *S*. Typhimurium LT2 could be a result of the cargo in the membrane vesicles interacting with proteins involved with bacterial cell division.

**Table 2.** Mutations identified in *S*. Typhimurium LT2 mutants resistant to self-inhibitory effects of membrane vesicle-enriched secretomes.

Strain	Resistant to vesicle-enriched secretome isolated from	Mutation (amino acid change)
DA69591	*S*. Typhimurium LT2	2-fold amplification in genomic regions with coordinates 544506–786683
DA69593	*S*. Typhimurium LT2	2-fold amplification in genomic regions with coordinates 530416–647413
DA69594	*S*. Typhimurium LT2	*ftsW* (Leu186Pro)

### VES-mediated microbial interactions are dependent upon the type of nutrients in the environment

To determine how microbial interactions caused by the VES vary with a change in the type of nutrients in the environment, VESs of bacteria grown in nutrient-poor glucose minimal media were investigated for their effect on the growth of other bacterial species. Thus, VESs were isolated from *E. coli* ECOR 36, *S. arizonae* and *P. aeruginosa* PA14 after growth in M9-glucose minimal medium, and their effect was studied on growth of *E. coli* K-12 MG1655 and *S*. Typhimurium LT2. These sets of bacterial combinations were specifically chosen since under nutrient-rich conditions (i.e. in LB), VESs from *E. coli* ECOR 36 *and P. aeruginosa* PA14 had the strongest effect on *E. coli* K-12 MG1655 (Fig. S1D and H), while vesicle-enriched secretome from *S. arizonae* and *P. aeruginosa* (PA14) displayed a strong effect on *S*. Typhimurium LT2 (Fig. S1F and H).

**Figure 6.**
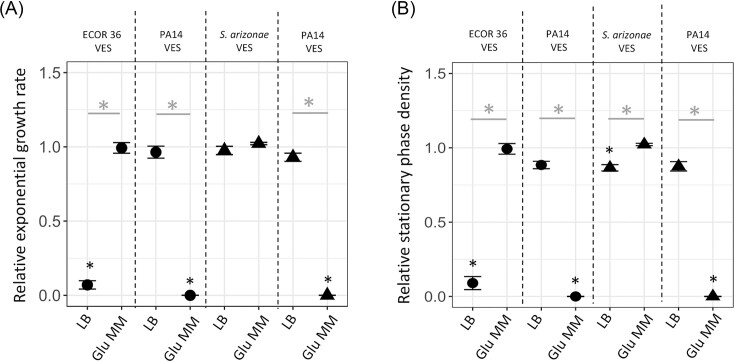
Dependence of antagonistic effects of vesicle-enriched secretomes (VESs) on the nutrients in the environment. (A) Relative exponential growth rate and (B) relative stationary phase density for *E. coli* K-12 MG1655 (circles) and *S*. Typhimurium LT2 (triangles) measured under different growth conditions in the presence of VESs isolated from ECOR 36, *S. arizonae* and *P. aeruginosa* PA14. The different growth conditions used were either nutrient-rich lysogeny broth (LB) or nutrient-poor glucose (0.2%) M9- minimal media (Glu MM). All growth parameters are shown normalized to values obtained for the ancestral strain grown in the absence of the VES. A two-sided Student's-t test was performed to determine statistical significance. * indicates statistical significance at *P* < 0.001 when growth parameters are compared to parameters of ancestral strain grown in the absence of VESs, while * indicates statistical significance when the normalized parameters are compared across conditions. Error bars represent the standard deviation in each case. The concentration of the VESs is mentioned in Table S2.

Overall, the growth medium had a strong effect on VES-mediated microbial interactions (Fig. 6). VESs isolated from ECOR 36 had no effect on either the exponential growth rate or the stationary phase density of *E. coli* K-12 MG1655 in the nutrient-poor glucose minimal medium, as compared to the nutrient-rich LB medium where it had strong effects on both these growth parameters. Similarly, the VESs of *S. arizonae* grown on nutrient-poor medium had no effect on the growth of *S*. Typhimurium LT2, instead of the antagonistic effect that was observed under nutrient-rich conditions (Fig. 6 and Tables S3 and S4, relative stationary phase density statistically different at *P* < 0.001 for a two-sided Student's t-test). On the other hand, vesicles from *P. aeruginosa* had a much stronger antagonistic effect on both *E. coli* K-12 MG1655 and *S*. Typhimurium LT2 in nutrient-poor medium as compared to nutrient-rich LB medium (Fig. 6 and Tables S3 and S4, statistically different at *P* < 0.001 for a two-sided Student's t-test), such that the bacteria displayed no growth at all in the presence of the VES in the former medium. Although our results conclusively show that VES-mediated microbial interactions are dependent on the type of nutrient in the environment, more work is needed to understand whether this is due to a change in the properties of the vesicles, or due to a change in physiology of the bacteria itself, or both.

## Discussion

Membrane vesicles and vesicle-enriched secretomes have mainly been studied due to their potential medical significance, either as carriers of virulence factors (Kesty et al. 2004, Kaparakis-Liaskos and Ferrero 2015, Koeppen et al. 2016, Nice et al. 2018) or as vaccine delivery vehicles (van der Pol et al. 2015, Cheng et al. 2021), and it is only recently that their ecological relevance and importance in bacterial communities have been examined. Our results show that VESs can indeed have strong effects on microbial community composition by altering the competitive abilities of the bacteria present. Thus, in the two- and three-species systems examined here, the addition of VESs from the bacterial species that was a weak competitor resulted in its maintenance in the community, which was in part due to the antagonistic effects of VESs toward other bacterial species. Furthermore, using an experimental evolution approach, we showed that these VESs may act as selective pressures that influence the composition of the microbial community.

### Significance of membrane VESs as antagonistic agents in microbe-microbe interactions

VESs carry different types of proteins that can influence the growth of both the producing and neighboring bacterial species. We observed several cases of antagonistic effects of VESs, and in one case demonstrated that the observed antagonism was due to colicins, a group of ecologically relevant toxins. Thus, the vesicle-enriched secretomes of all the natural isolates of *E. coli* that contained a colicin-producing plasmid displayed antagonism against *E. coli* K-12 MG1655. Colicins are generally secreted as soluble proteins in the bacterium's environment; thus in their soluble state, they would not be co-isolated with the membrane vesicles using the method we have used to enrich vesicles. Thus, this observation suggests a novel association between colicins and membrane vesicles. Interestingly, the antagonistic effect observed from these VES was dependent on the genotype of the colicin-producing bacteria, such that the bacteria secreting the same type of colicin exhibited different levels of growth inhibition. Although this could either be due to different levels of colicin being expressed, different amounts of vesicles being secreted, or different levels of association between vesicles and colicins among different isolates, this result highlights the important role of the bacterial genotype in effecting the properties of VESs.

Antagonistic effects were also observed for the VESs isolated from *P. aeruginosa* PA14. Membrane vesicles from *P. aeruginosa* have been extensively studied, and are known to carry compounds that can affect both eukaryotic and prokaryotic cells. These studies have shown that microbial interactions mediated by the vesicles isolated from *P. aeruginosa* are largely observed due to the association of antibacterial quinolones, iron-scavenging proteins, autolysin enzymes, and quorum-sensing inducers with the vesicles (Kadurugamuwa and Beveridge 1996, Tashiro et al. 2012, Tashiro et al. 2013, Cooke et al. 2019). Our results corroborate the observations from earlier studies in demonstrating the antagonistic effects of membrane vesicles from *P. aeruginosa* on a variety of bacterial species.

### Environments containing different nutrient alter VES-mediated microbial interactions

For a given species of bacteria, the number of vesicles produced and the cargo present within these vesicles can vary with growth conditions (Macdonald and Kuehn 2013, Orench-Rivera and Kuehn 2016). For example, membrane vesicles isolated from *Pseudomonas putida* KT2440 grown in benzoate-containing minimal media contained benzoate-degrading enzymes which were absent in membrane vesicles when the bacteria were grown in nutrient-rich lysogeny broth (Choi et al. 2014). Several studies have also shown increased vesicle production under stressful conditions, which is either a defense mechanism against these stresses or an indirect effect of other stress response pathways. Important roles of membrane vesicles have been suggested in maintaining homeostasis under conditions leading to membrane stress, protein misfolding, and nutrient starvation (Nagakubo et al. 2019). Besides this, the presence of antibacterial agents, compounds that affect the pH of the system, the presence of phages, and non-optimal temperatures may increase vesiculation in bacteria (Manning and Kuehn 2011, de Jonge et al. 2021), implying that microbial interactions that are mediated through vesicle-enriched secretomes also depend on the environment. Results from this study support this assertion, where two instances of VESs-mediated interactions changed with alterations in the environmental conditions. Thus, the VES isolated from *P. aeruginosa* PA14 showed different levels of antagonism towards *E. coli* K-12 MG1655 and *S*. Typhimurium LT2 based on nutrient levels, whereas the effects of VES isolated from *S. arizonae* on growth of *S*. Typhimurium went from being antagonistic under nutrient-rich conditions to having no effect under nutrient-poor conditions. These results highlight how the type of nutrient in an environment can influence the effect of the VESs on microbial community dynamics.

### VESs can result in self-inhibition of the producing species

We also observed self-inhibitory effects of the vesicle-enriched secretomes, where the vesicle-enriched secretomes isolated from *S*. Typhimurium, *E. coli* ECOR 36, and *S. arizonae* resulted in self-inhibition. Although the ecological or evolutionary relevance of self-inhibition by vesicles is unclear, our work demonstrates that mutants that are resistant to such self-inhibitory effects could rapidly be selected since resistant *S*. Typhimurium LT2 mutants emerged in presence of its own vesicle-enriched secretome. One of these resistant mutants had a mutation in a cell-division protein FtsW, suggesting that the self-inhibiting effects might be due to the interaction of the vesicle-enriched secretome with the cell-division machinery.

## Conclusion

Membrane vesicle production is a common phenotype observed in all bacteria and by tying bacterial fitness to membrane vesicle production, our study has taken an initial step in elucidating the mechanisms by which vesicle-enriched secretomes can affect community dynamics. To demonstrate these effects, we have used vesicle-enriched secretomes where the vesicles were concentrated ∼ 8-fold. It is notable that several mutations have been shown to individually increase the level of vesicle production (Kulp et al. 2015). Consequently, an important next step to understand the relevance of these vesicles in microbial communities would be to identify ecological conditions where mutations that change characteristics (amount, composition) of the vesicle-enriched secretome are enriched by selection. Such studies will further increase our understanding of the evolutionary and ecological significance of these vesicles.

## Supplementary Material

fiad141_Supplemental_Files

## Data Availability

The whole-genome sequence files (fastq) used in this study are deposited at NCBI's sequence read archive (SRA) under the BioProject ID PRJNA1030170 with BioSample accession numbers from SAMN37890565 to SAMN37890570.
