# Supplementary material for: Vesicle-enriched secretomes alter bacterial competitive abilities and are drivers of evolution in microbial communities
Source: FEMS Microbiol Ecol. 2023 Oct 26;99(12):fiad141. doi: 10.1093/femsec/fiad141 (PMC10653989; doi:10.1093/femsec/fiad141)

Supplementary Fig. 1. Effect of vesicle-enriched secretomes (VESs) isolated from A) *E. coli* K-12 MG1655 B) *E. coli* ECOR 5 C) *E. coli* ECOR 11 D) *E. coli* ECOR 36 E) *S.* Typhimurium LT2 F) *S.arizonae* G) *P. aeruginosa* PA01 H) *P.aeruginosa* PA14 I) *B.subtilis* on relative exponential growth rate and relative stationary density of different bacterial species when grown in LB. All values are normalized to growth parameters measured in the absence of VESs. Two-sided Students-t test was performed to determine statistical significance and * indicates statistical significance at p<0.001 when growth parameters are compared to parameters of ancestral strain grown in the absence of VES. Error bars represent the standard deviation in each case. Two VES preparations isolated separately were mixed together in each case, and the concentration of the VESs are mentioned in Supplementary table 2.

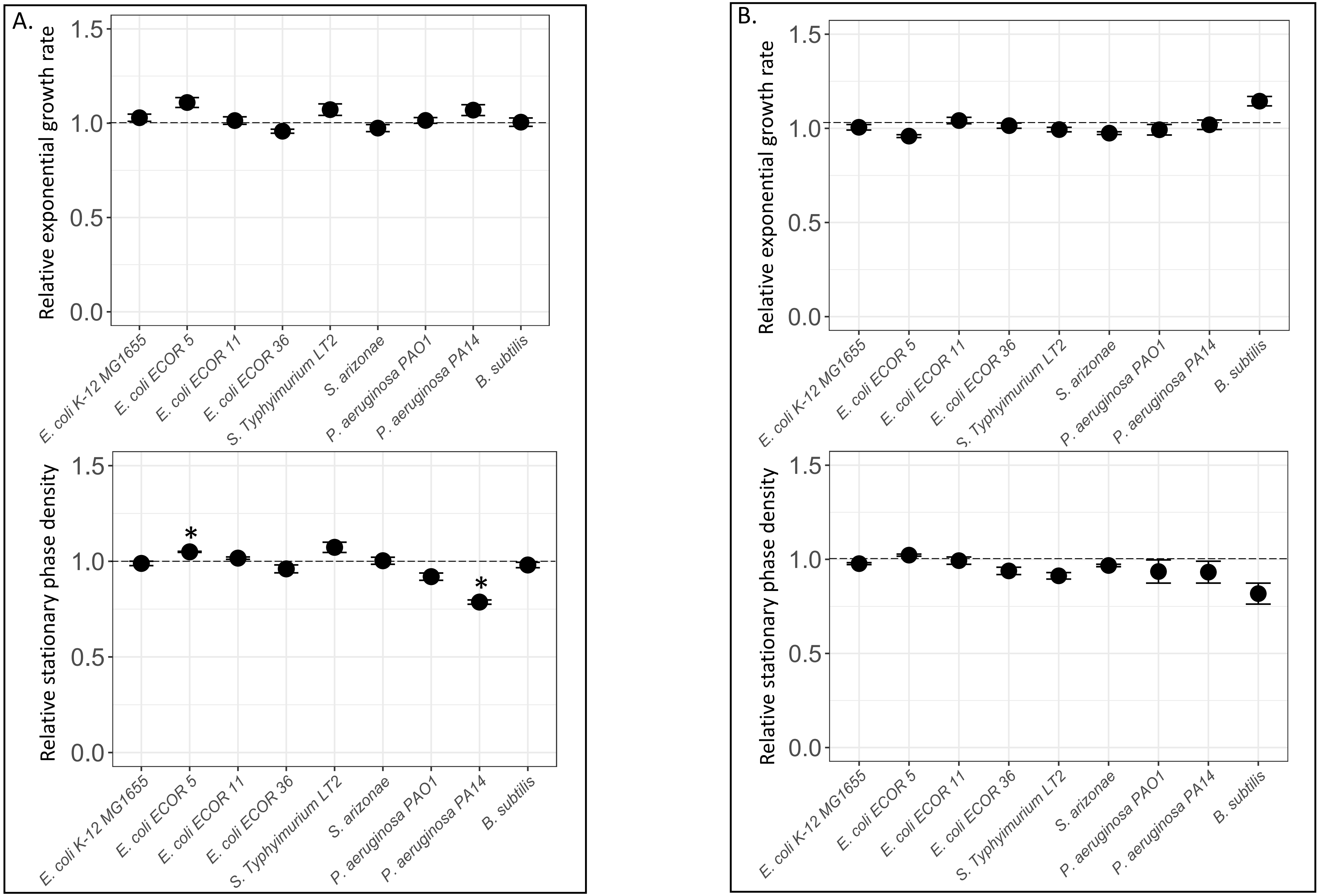

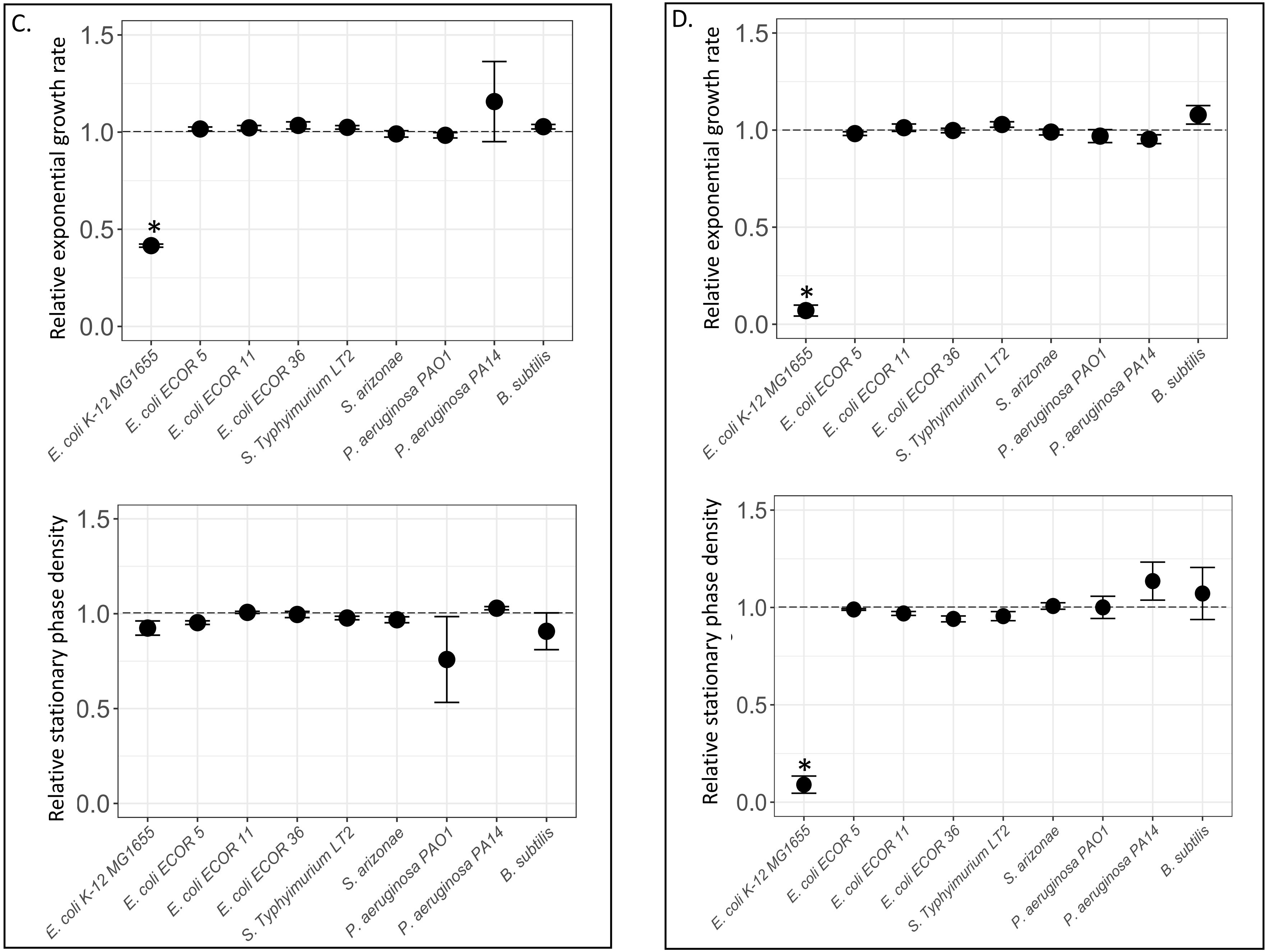
C.
Relative exponential growth rate
1.5
1.0
0.5
0.0
*
E. coli K-12 MG1655
E. coli ECOR 5
E. coli ECOR 11
E. coli ECOR 36
S. Typhyimurium LT2
S. arizonae
P. aeruginosa PAO1
P. aeruginosa PA14
B. subtilis
Relative stationary phase density
D.
Relative exponential growth rate
Relative stationary phase density

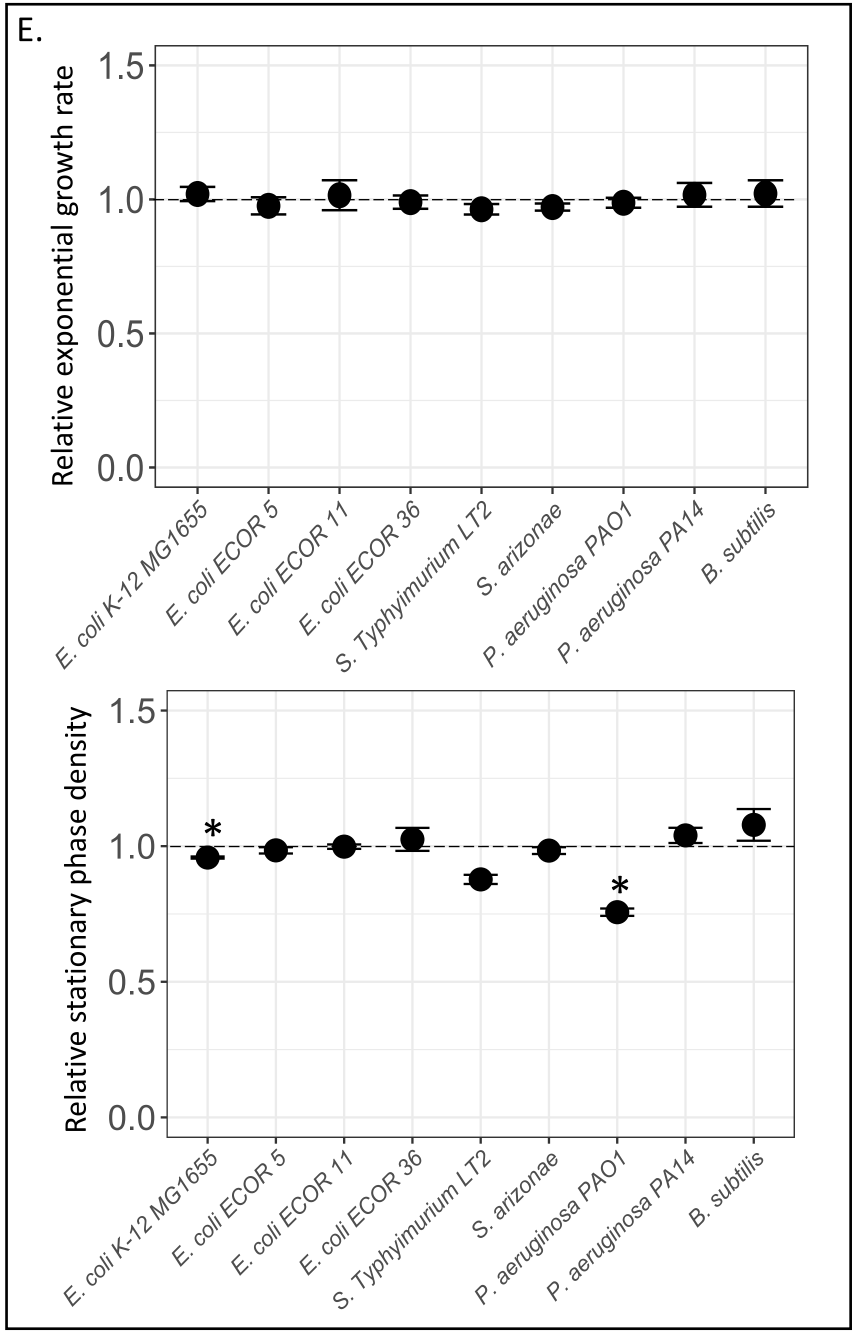

E.
Relative exponential growth rate
1.5
1.0
0.5
0.0
E. coli K-12 MG1655
E. coli ECOR 5
E. coli ECOR 11
E. coli ECOR 36
S. Typhyimurium LT2
S. arizonae
P. aeruginosa PAO1
P. aeruginosa PA14
B. subtilis
Relative stationary phase density
*

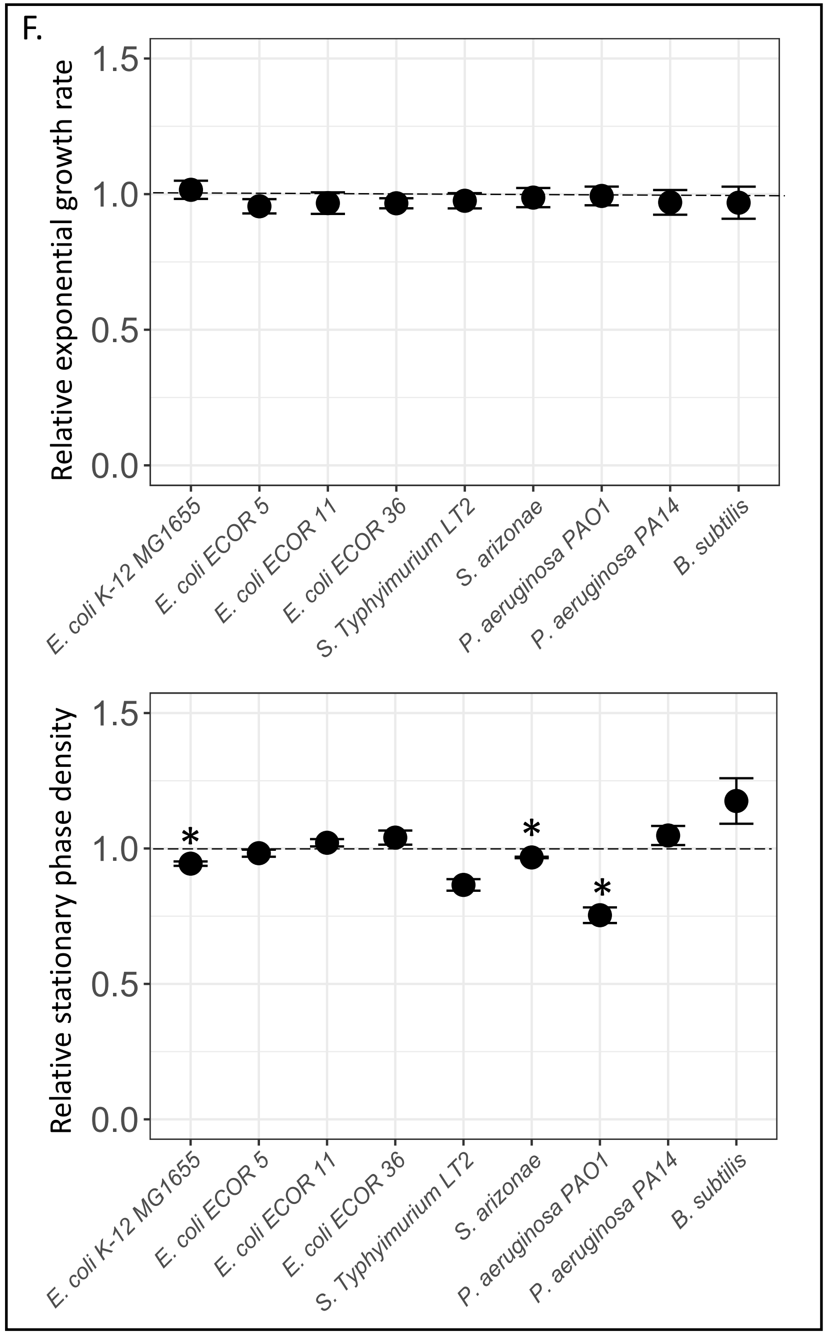

F.
Relative exponential growth rate
1.5
1.0
0.5
0.0
E. coli K-12 MG1655
E. coli ECOR 5
E. coli ECOR 11
E. coli ECOR 36
S. Typhyimurium LT2
S. arizonae
P. aeruginosa PAO1
P. aeruginosa PA14
B. subtilis
Relative stationary phase density
*

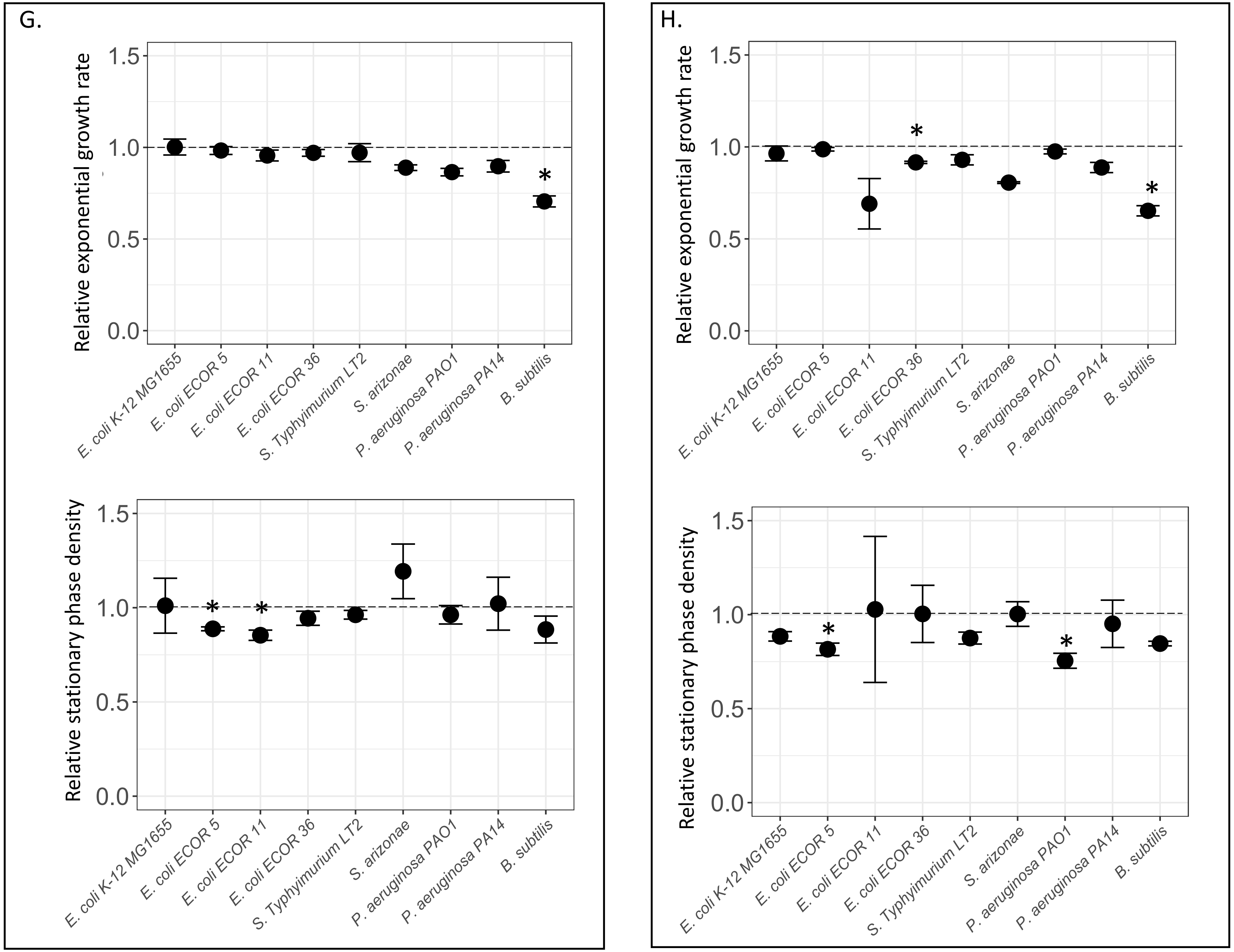
G.
H.
Relative exponential growth rate
Relative stationary phase density
1.5
1.0
0.5
0.0
*
E. coli K-12 MG1655
E. coli ECOR 5
E. coli ECOR 11
E. coli ECOR 36
S. Typhyimurium LT2
S. arizonae
P. aeruginosa PAO1
P. aeruginosa PA14
B. subtilis

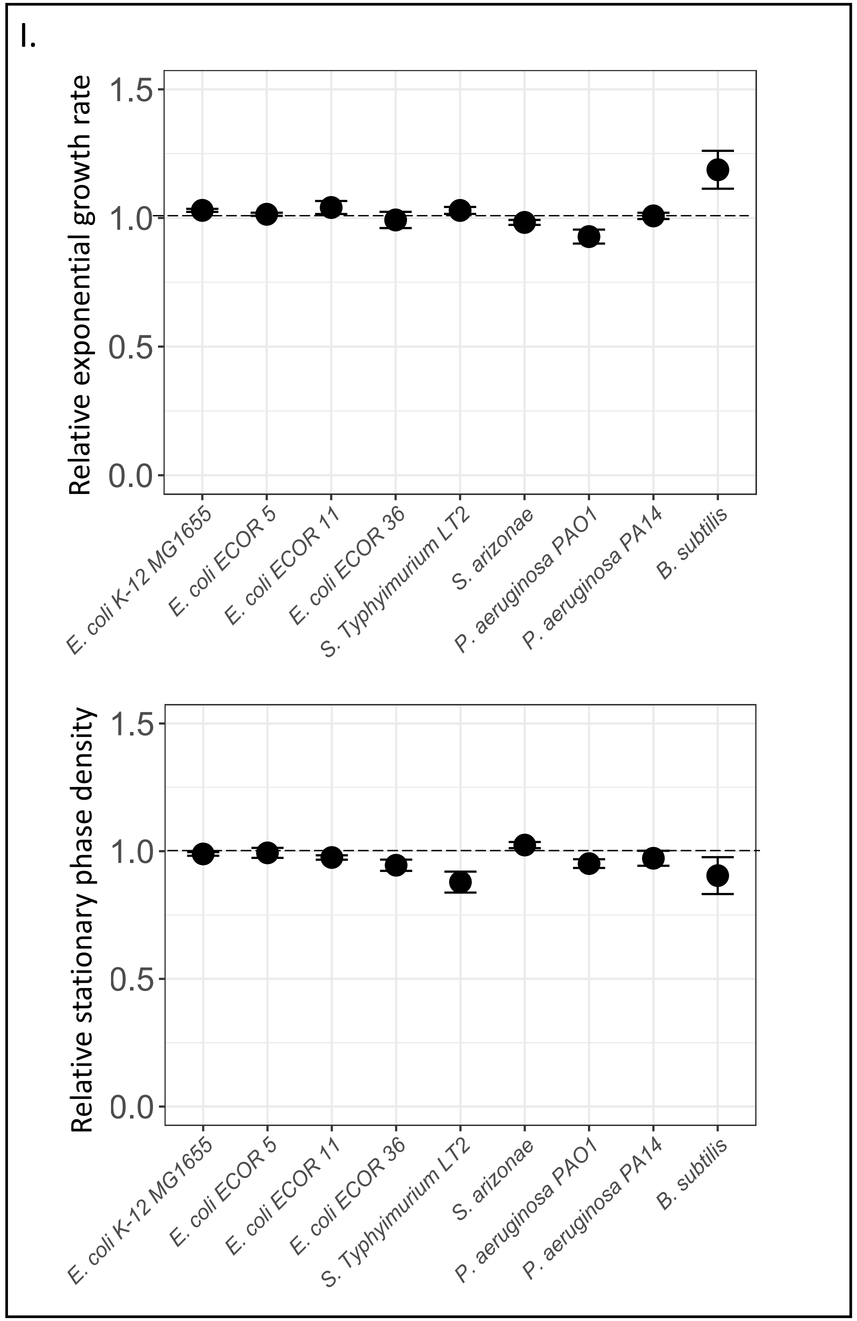
I.
Relative exponential growth rate
1.5
1.0
0.5
0.0
E. coli K-12 MG1655
E. coli ECOR 5
E. coli ECOR 11
E. coli ECOR 36
S. Typhyimurium LT2
S. arizonae
P. aeruginosa PAO1
P. aeruginosa PA14
B. subtilis
Relative stationary phase density
1.5
1.0
0.5
0.0
E. coli K-12 MG1655
E. coli ECOR 5
E. coli ECOR 11
E. coli ECOR 36
S. Typhyimurium LT2
S. arizonae
P. aeruginosa PAO1
P. aeruginosa PA14
B. subtilis

Supplementary Fig. 2. Reduced susceptibility for *E. coli* K12 MG1655 towards vesicle-enriched secretome isolated from ECOR 36 when *E. coli* K-12 MG1655 expresses the ColE1 immunity protein (triangle) as compared to parental *E. coli* K-12 MG1655 carrying empty plasmid (circle). Students-t test was performed to determine statistical significance and * indicates statistical significance at p<0.001 when growth parameters are compared to parameters of *E. coli* K-12 MG1655 carrying empty plasmid. Error bars represent the standard deviation in each case. Two VES preparations isolated separately were mixed together in each case, and the concentration of the VESs are mentioned in Supplementary table 2.

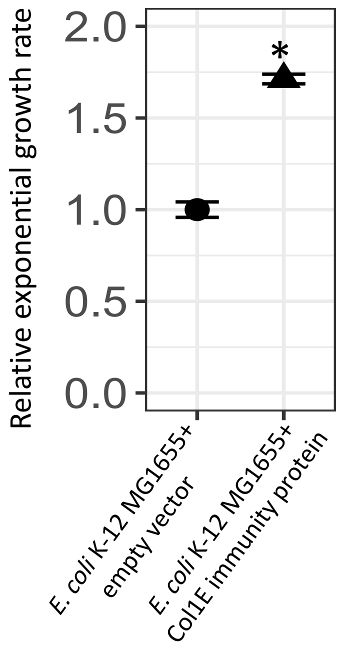

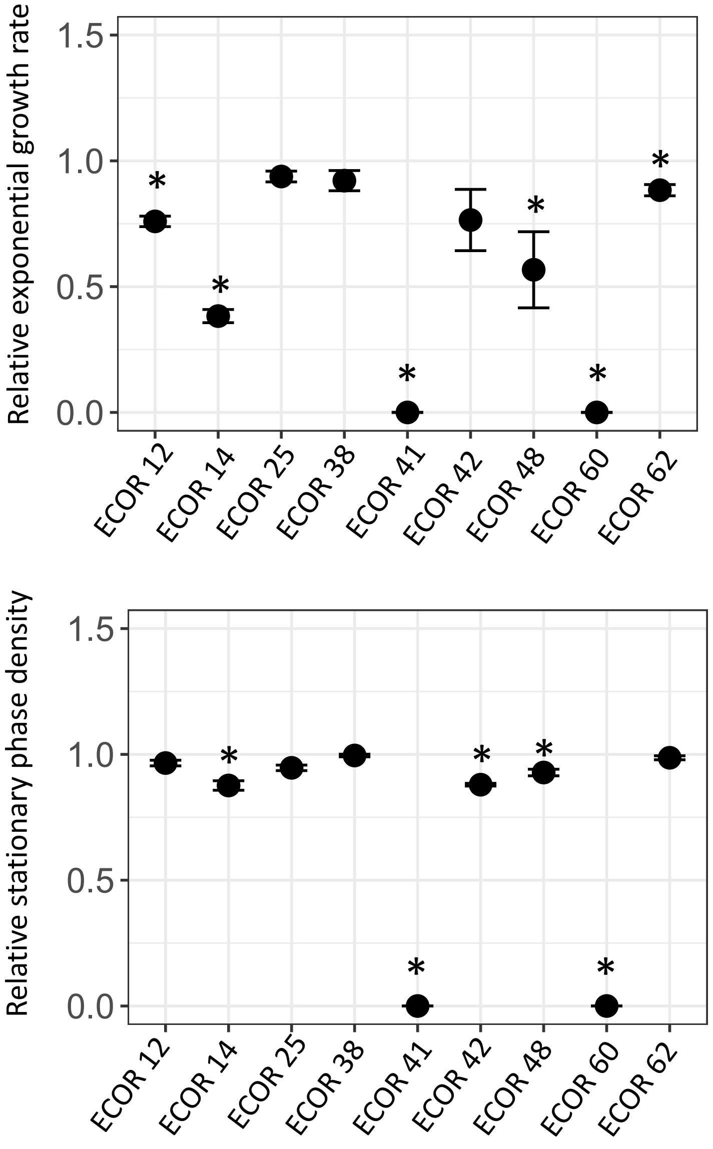

Supplementary Fig. 3. Association of vesicles-enriched secretomes (VES) with different types of colicins. Effect of vesicle-enriched secretomes isolated from known colicin-producing ECOR strains on exponential growth rate and stationary phase density of *E. coli* K-12 MG1655. All growth parameters are shown normalized to values obtained for the susceptible ancestral strain grown in the absence of the VESs. Four biological replicates were used in each case. Students-t test was performed to determine statistical significance and * indicates statistical significance at $p<0.001$ (corrected for multiple testing using Bonferroni's correction) when comparing the parameters measured in the absence and presence of VESs. Error bars represent the standard deviation in each case. Two VES preparations isolated separately were mixed together in each case, and the concentration of the VESs are mentioned in Supplementary table 2.

Supplementary Fig. 4. Host-dependent antagonistic effects of vesicle-enriched secretomes (VESs). Different levels of antagonism were observed from vesicle-enriched secretomes isolated from colicin B producing *E. coli* strains (circles) and colicin E1 producing *E. coli* strains (triangles) on A) exponential growth rate of *E. coli* K-12 MG1655 B) stationary phase density of *E. coli* K-12 MG1655. A one-way ANOVA was done to determine statistical significance. * indicates statistical significance at p<0.01. F-values, p-values for one-way ANOVA and adjusted p-values for Tukey's HSD given in Supplementary table 3 and 4 Error bars represent the standard deviation in each case. Two VES preparations isolated separately were mixed together in each case, and the concentration of the VESs are mentioned in Supplementary table 2.

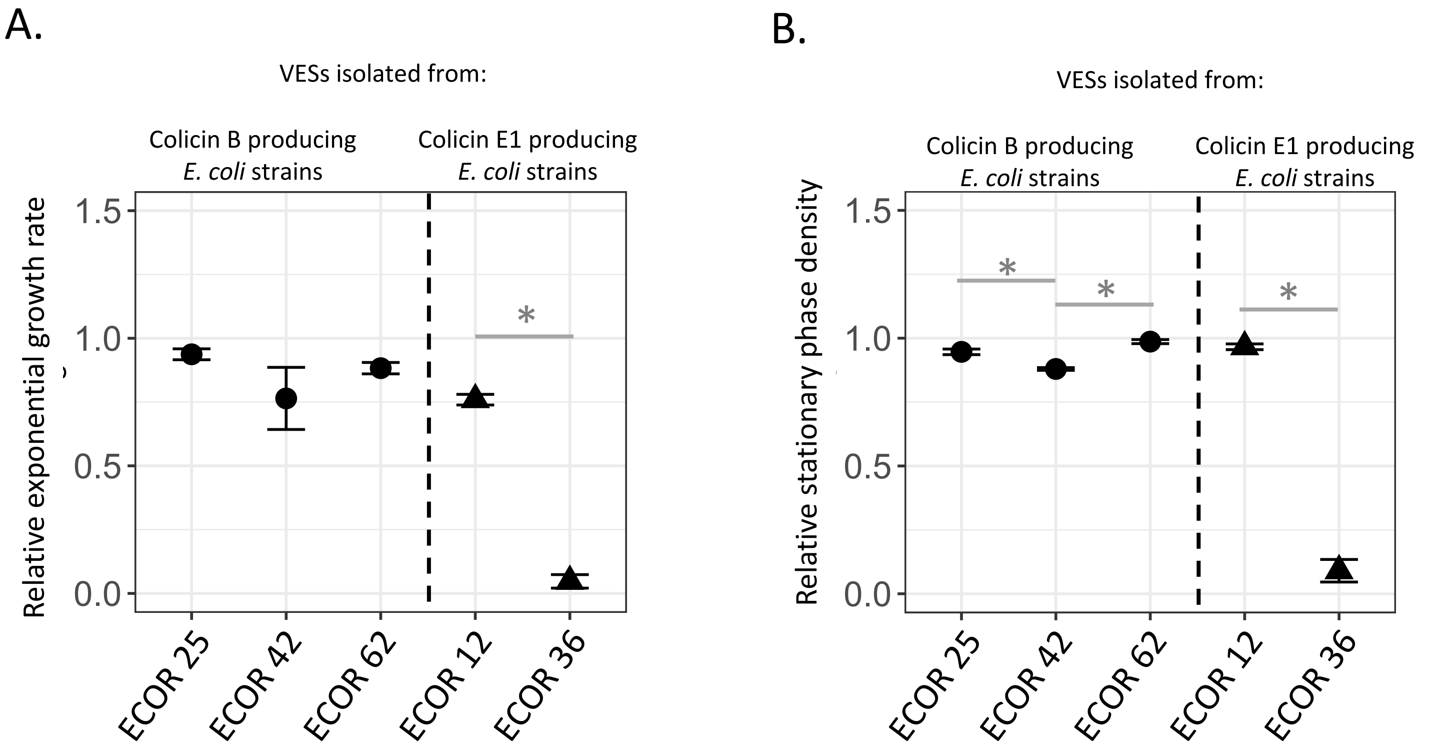

Supplementary Fig. 5. Cryo-EM images of VESs isolated from *E. coli* ECOR36. The presence of membrane vesicles in the vesicle-enriched secretomes of ECOR36 was demonstrated by performing cryo-EM investigation.

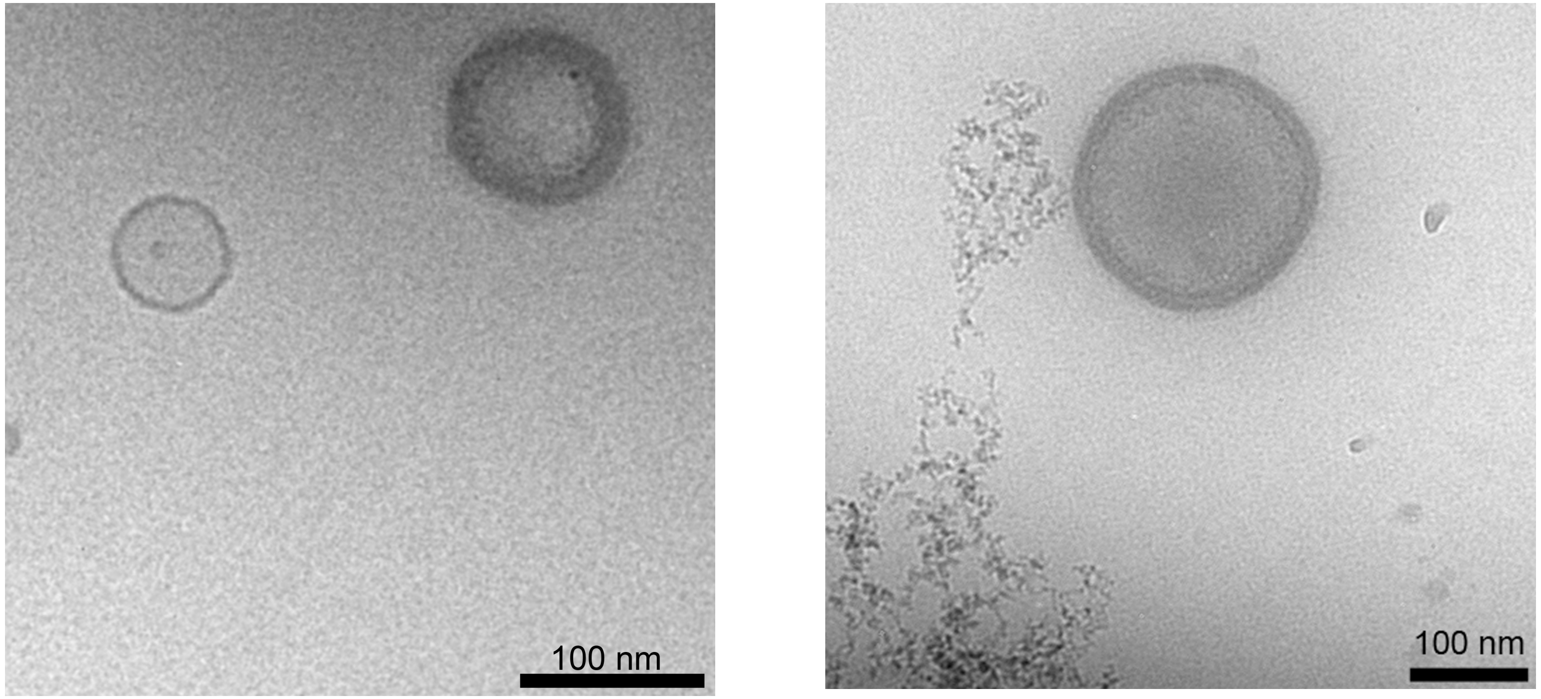

Supplement: fiad141_Supplemental_Files [file fiad141_supplemental_files.zip › Warsi_et_al._Supp_data figures.pdf]
