# Supplementary figures and images for: Vesicle-enriched secretomes alter bacterial competitive abilities and are drivers of evolution in microbial communities
Source: FEMS Microbiol Ecol. 2023 Oct 26;99(12):fiad141. doi: 10.1093/femsec/fiad141 (PMC10653989; doi:10.1093/femsec/fiad141)

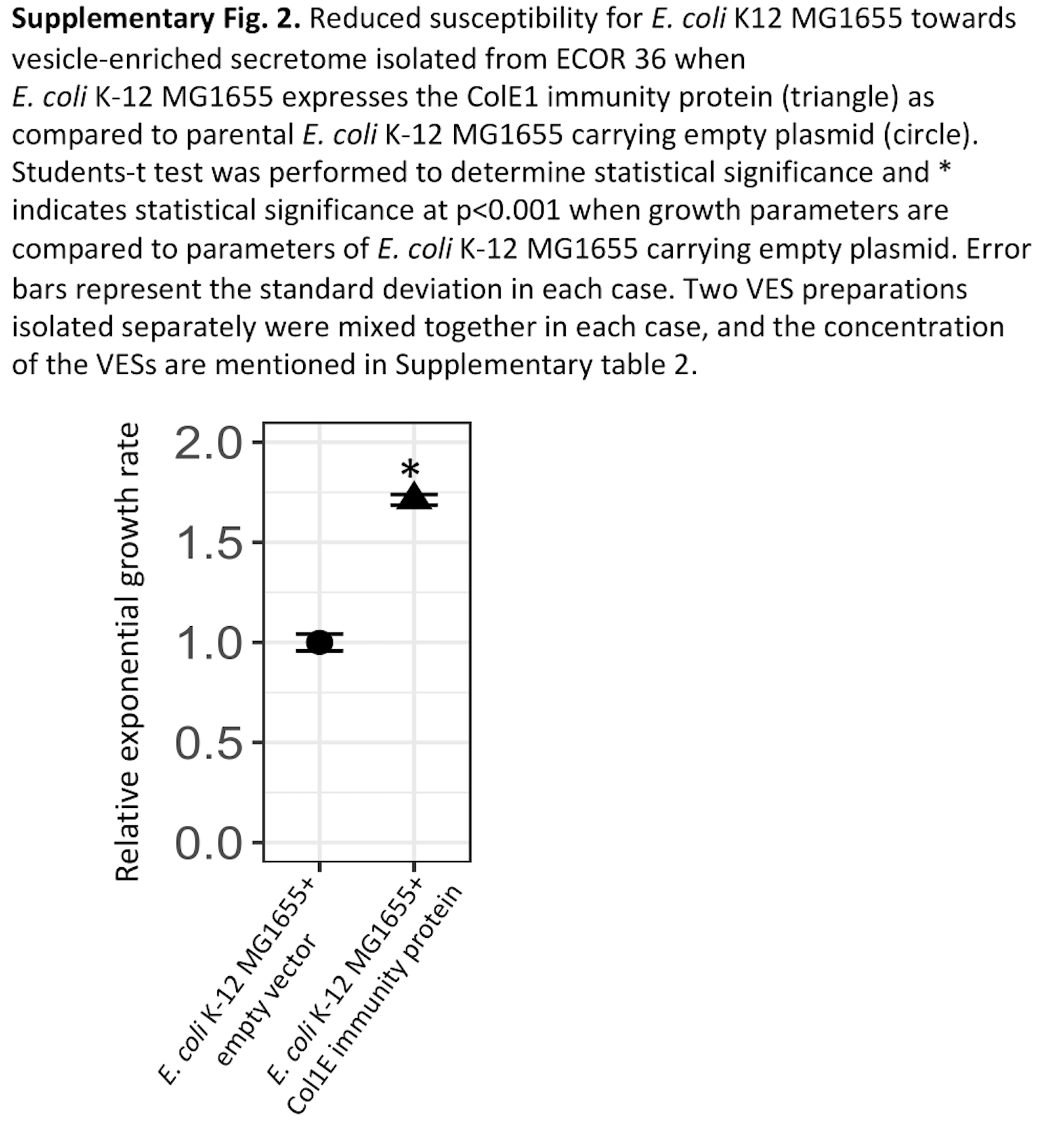

Supplement: fiad141_Supplemental_Files [file fiad141_supplemental_files.zip › Supp_data fig. 2.tiff]

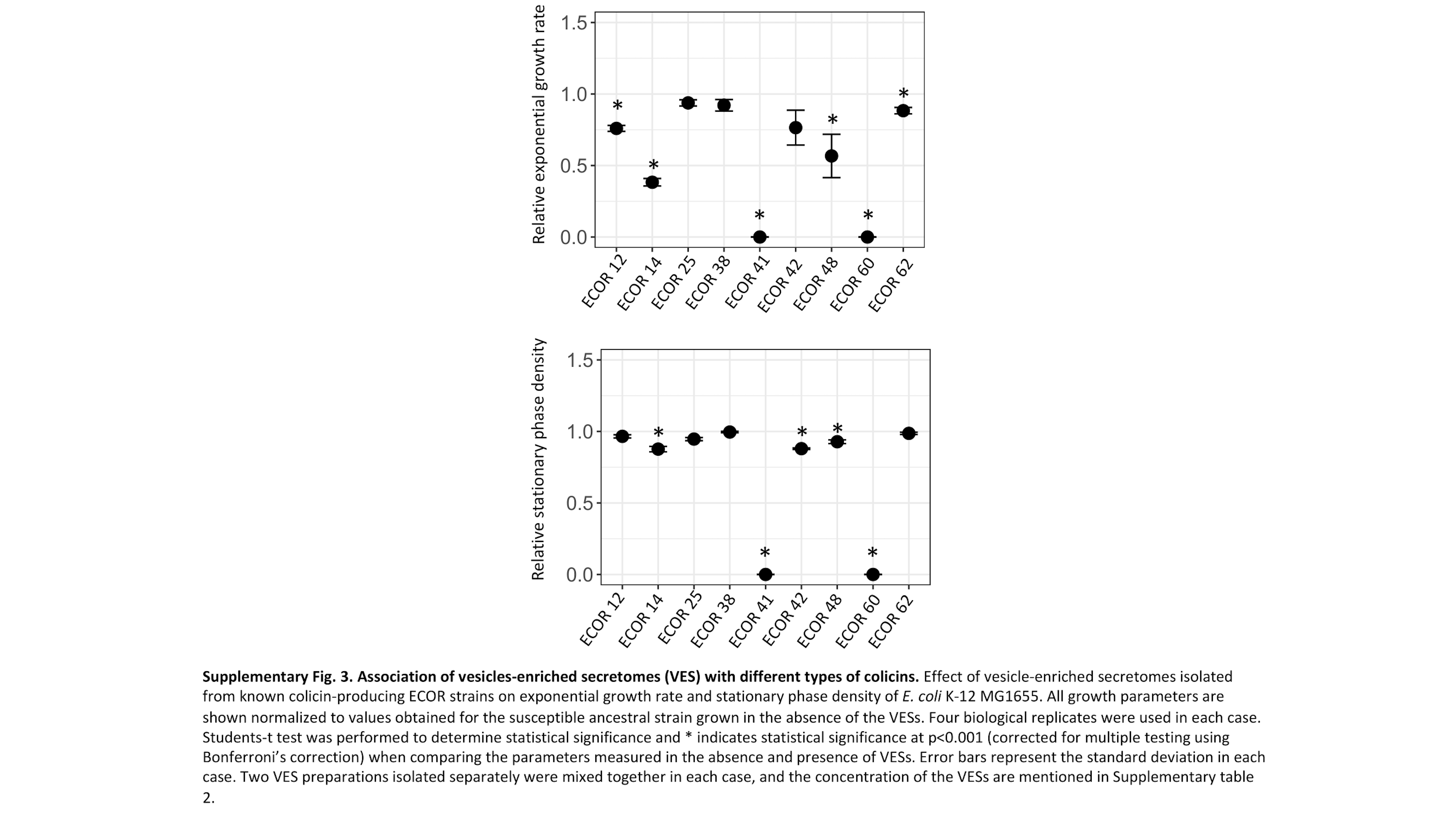

Supplement: fiad141_Supplemental_Files [file fiad141_supplemental_files.zip › Supp_data fig. 3.tiff]

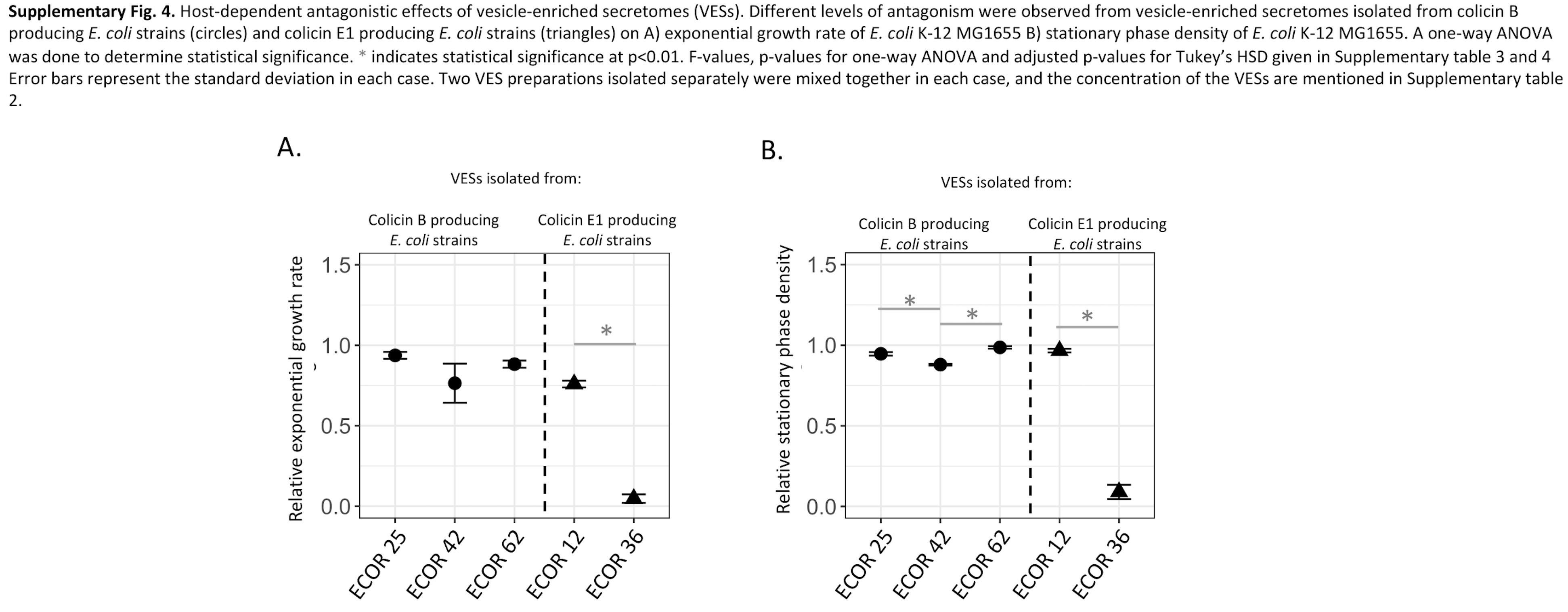

Supplement: fiad141_Supplemental_Files [file fiad141_supplemental_files.zip › Supp_data fig. 4.tiff]

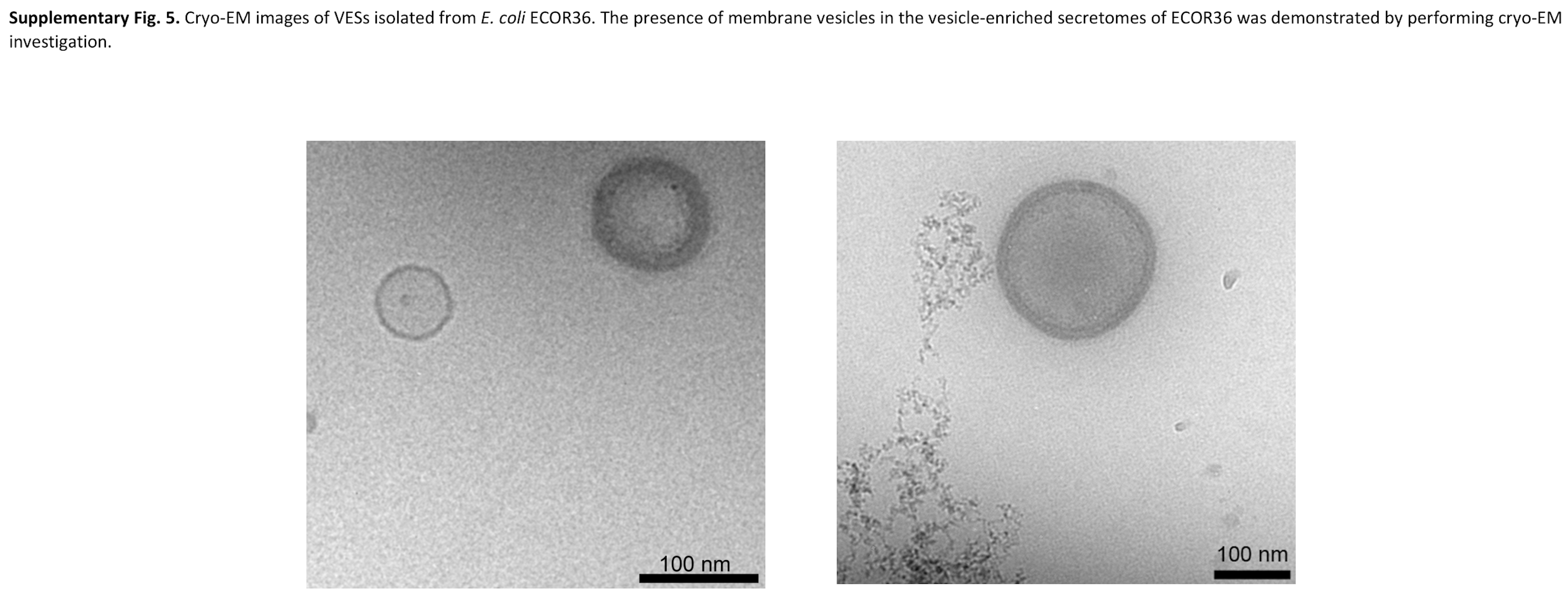

Supplement: fiad141_Supplemental_Files [file fiad141_supplemental_files.zip › Supp_data fig. 5.tiff]

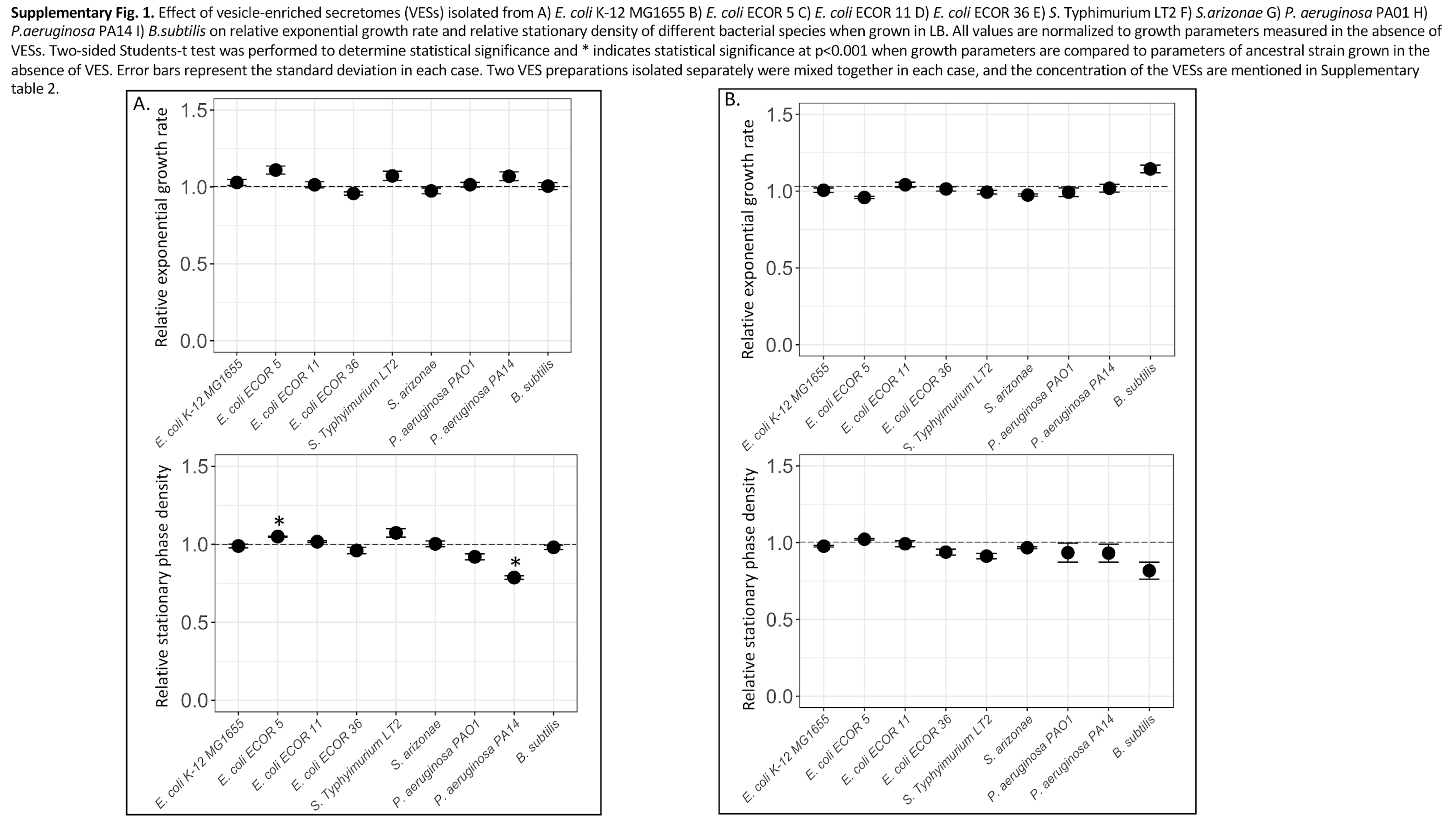

Supplement: fiad141_Supplemental_Files [file fiad141_supplemental_files.zip › Supp_data fig. 1.tiff]
